# Accelerated aging-related transcriptome changes in the female prefrontal cortex

**DOI:** 10.1111/j.1474-9726.2012.00859.x

**Published:** 2012-10

**Authors:** Yuan Yuan, Yi-Ping Phoebe Chen, Jerome Boyd-Kirkup, Philipp Khaitovich, Mehmet Somel

**Affiliations:** 1 Key Laboratory for Computational Biology, CAS-MPG Partner Institute for Computational Biology, Shanghai Institutes for Biological Sciences, Chinese Academy of Sciences320 Yue Yang Road, Shanghai 200031, China; 2 Department of Computer Science and Computer Engineering, La Trobe UniversityMelbourne, Vic. 3086, Australia; 3 Max Planck Institute for Evolutionary AnthropologyDeutscher Platz 6, D-04103 Leipzig, Germany; 4 Department of Integrative Biology, University of California–BerkeleyBerkeley, CA, USA

**Keywords:** Alzheimer’s disease, central nervous system, gene expression, prefrontal cortex, sex difference

## Abstract

Human female life expectancy is higher than that of males. Intriguingly, it has been reported that women display faster rates of age-related cognitive decline and a higher prevalence of Alzheimer’s disease (AD). To assess the molecular bases of these contradictory trends, we analyzed differences in expression changes with age between adult males and females, in four brain regions. In the superior frontal gyrus (SFG), a part of the prefrontal cortex, we observed manifest differences between the two sexes in the timing of age-related changes, *that is,* sexual heterochrony. Intriguingly, age-related expression changes predominantly occurred earlier, or at a faster pace, in females compared to men. These changes included decreased energy production and neural function and up-regulation of the immune response, all major features of brain aging. Furthermore, we found that accelerated expression changes in the female SFG correlated with expression changes observed in AD, as well as stress effects in the frontal cortex. Accelerated aging-related changes in the female SFG transcriptome may provide a link between a higher stress exposure or sensitivity in women and the higher prevalence of AD.

## Introduction

Human males and females display subtle dimorphism in their rates of brain development and brain anatomy [reviewed in (Lenroot & Giedd, 2010; Joel, 2011)]. The question of whether the two sexes differ in their rates of brain aging, however, is unclear. On the one hand, female life expectancy is ∼5 years greater than that of males (Møller *et al.*, 2009). Women are also less affected by particular aging-related neurodegenerative disorders, including Parkinson’s (Miller & Cronin-Golomb, 2010) and Huntington’s disease (Bode *et al.*, 2008). These observations suggest slower female brain aging, *that is,* slower age-related functional decline and lower propensity to disease in females. In contrast, multiple studies have reported a higher age-specific risk of dementia and Alzheimer’s disease (AD) in women (Andersen *et al.*, 1999; von Strauss *et al.*, 1999; Barnes *et al.*, 2005; Corrada *et al.*, 2008; Schmidt *et al.*, 2008) [note, however, that not all studies have detected this difference; *e.g.* (Kawas *et al.*, 2000; Katz *et al.*, 2011)]. For example, studying 911 individuals above 90 years of age, (Corrada *et al.*, (2008) found that the incidence of dementia doubled every 5 years among women, but not among men. This implies faster deterioration of particular processes in the aging female brain and is reminiscent of the ‘health-survival paradox’: old women appear in worse health than old men, but men exhibit higher mortality (Oksuzyan *et al.*, 2008). One biological explanation for these contradictory patterns is that different brain tissues or physiological processes have different aging rates between sexes: some deteriorate faster in males, others, in females. Such dimorphism in brain aging rates and its heterogeneity among tissues, however, has not yet been documented.

One approach that can be used to investigate rates of age-related functional decline is transcriptome analysis. Brain gene expression levels change significantly over lifetime, from birth till old age, and these changes follow distinct and evolutionarily conserved trajectories that reflect functional changes in the tissue (Lee *et al.*, 2000; Lu *et al.*, 2004; Somel *et al.*, 2010). But the pace of these age-related expression changes varies among organisms, depending on genotype and environment, a phenomenon termed ‘transcriptional heterochrony’ (Zakany *et al.*, 1997). By analyzing rates of expression changes with age, one can thus infer relative rates of development or aging-related functional decline in different organisms.

Here, we use brain gene expression age-series to investigate possible differences in aging rate between sexes. We start by reanalyzing a human brain age-series dataset comprising four brain regions (Berchtold *et al.*, 2008). In the original study, the authors identified prominent expression differences between sexes during brain aging, such as stronger down-regulation of protein synthesis and energy pathways in male aging, but possible sexual heterochrony in brain aging was not tested directly. Applying an algorithm developed in our group, significance dynamic time warping (DTW-S) (Yuan *et al.*, 2011) on this dataset, we find an unexpected expression pattern in the prefrontal cortex: earlier or faster aging-related expression changes in females relative to males, implying faster functional deterioration in women. Analyzing the potential causes and consequences of this pattern, we find that it is connected to stress- and AD-related expression changes in the prefrontal cortex.

## Results

We utilized a published microarray time series containing gene expression profiles from cognitively healthy adult males and females (Berchtold *et al.*, 2008) (DATASET1 in Table S1). The sample size was ∼20 individuals per sex, and subject ages ranged from 20 to 99 years (Fig. S1). Expression was measured in four brain regions: the superior frontal gyrus (SFG), part of the prefrontal cortex; the postcentral gyrus (PCG), part of the somatosensory cortex; the hippocampus (HC), involved in long-term memory formation; and the entorhinal cortex (EC), which connects the HC with neocortical areas (Berchtold *et al.*, 2008). Individual sex identity was confirmed based on sex chromosomal expression patterns (Methods).

### Sexually dimorphic gene expression patterns

**Fig. 1:**
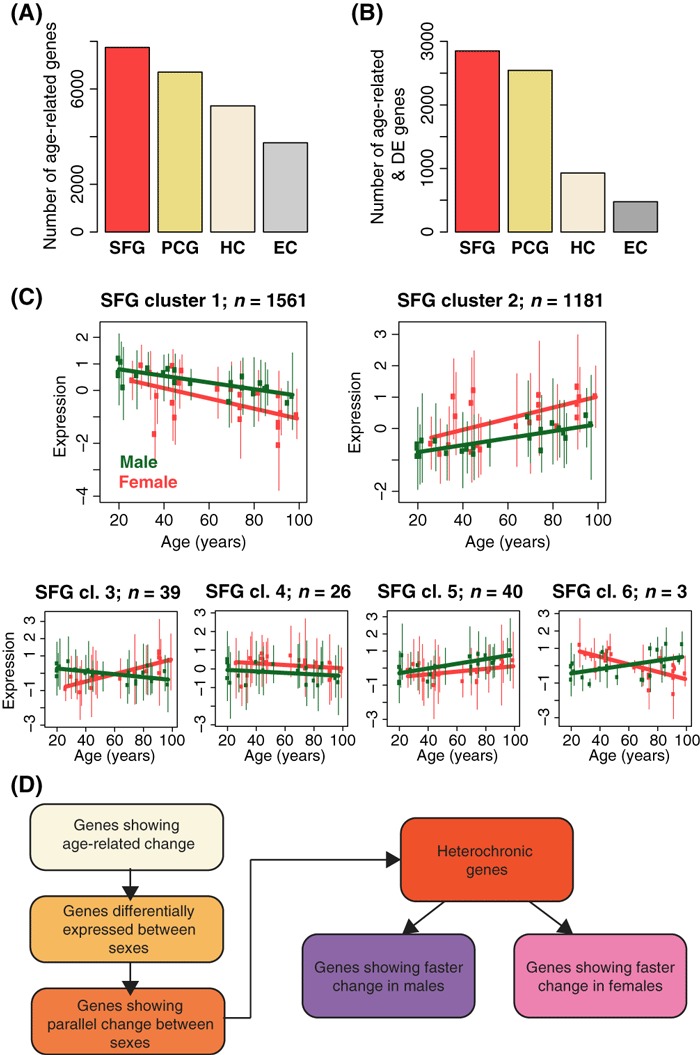
Sexually dimorphic gene expression changes in human brain aging. (A) The number of significantly age-related genes in each of the four brain regions. (B) The number of significantly age-related and significantly differentially expressed (DE) genes between two sexes, in each brain region. (C) Genes showing both significant age-related change and significant differential expression between sexes in SFG clustered into six groups using nonsupervised hierarchical clustering. *x*-axis: age in years; *y*-axis: male (green) and female (red) SFG expression levels normalized to mean = 0, standard deviation = 1. Points represent the mean per individual among genes within a cluster; vertical lines indicate variation (5–95% range). The number above each panel indicates the number of genes within each cluster. Similar clusters constructed for PCG, HC, and EC are shown in Fig. S2. (D) Schema describing the steps used to identify sexual heterochrony. Each box corresponds to a set of genes defined by a particular test (age-related change: multiple regression; differential expression: ancova; parallel changes: Pearson correlation; heterochrony: DTW-S). For the numbers of genes passing each step, see Table S2.

Among >13 000 expressed genes, we first identified genes showing significant expression changes with age within each of the four brain regions, using polynomial regression (Somel *et al.*, 2010) (Methods). Across the four regions, we found that 27–55% of genes showed age-related expression change (*F*-test *P* < 0.05; Fig. 1A, Table S2). Next, we tested each gene for expression divergence between sexes using analysis of covariance. Among the identified age-related genes, ∼2500 (37–38%) showed significant sex differences in the SFG and PCG, while <1000 (13–18%) showed significant differences in HC and EC (*F*-test *P* < 0.05; Fig. 1B, Table S2). Notably, the two neocortical regions, SFG and PCG, reveal a higher degree of age-related change, and a higher divergence between males and females, compared to the two allocortical regions, HC and EC – a pattern also detected in the original study (Berchtold *et al.*, 2008).

We then visualized age-related and differentially expressed genes by hierarchical clustering analysis (Figs 1C and S2). This revealed two notable patterns: first, in SFG and PCG, the majority of expression changes with age follow the same direction between males and females. In support of this, 87% (SFG), 83% (PCG), 63% (HC), and 40% (EC) of differentially expressed genes showed significant positive correlation between the two sexes’ expression-age trajectories (Pearson correlation test *P* < 0.05).

Second, the two largest gene clusters in both SFG and PCG (clusters 1 and 2 in Fig. 1C and in Fig. S2A) exhibited patterns indicative of sexual heterochrony, and in a particular direction. Namely, genes up-regulated during aging had higher average expression levels in females, thus they matched those of older males. Likewise, genes down-regulated during aging had lower expression in females. Both patterns are consistent with earlier or faster changes in females.

Meanwhile, in EC and HC, we found no comparable bias in the direction of heterochrony. In these brain regions, genes higher and lower expressed in one sex over the other were relatively evenly distributed among clusters (Fig. S2B–C). Moreover, in EC, genes in the largest clusters showed opposite trends of age-related change between males and females, such that sex differences cannot be attributed to heterochrony (Fig. S2C). Therefore, the trend toward faster age-related changes in females can only be detected in SFG and PCG clusters.

### Sexual heterochrony in prefrontal cortex expression

To formally test expression heterochrony, we took advantage of the DTW-S algorithm (Yuan *et al.*, 2011), which has been specifically designed for analyzing expression heterochrony in large-scale gene expression time series. The algorithm estimates the significance of measured heterochrony using a simulation-based test. As described in Fig. 1D, we applied DTW-S to genes showing (i) age-related change, (ii) differential expression between sexes, (iii) positive correlation between male and female trajectories (*n* = 2490 in SFG, *n* = 2102 in PCG, *n* = 584 in HC, and *n* = 186 in EC) (Table S2, Methods). Among these genes, we found 667 (27%) genes that showed significant heterochrony between sexes in SFG, while only 336 (16%) showed significant heterochrony in PCG, 81 (14%) in HC, and 35 (19%) in EC.

**Fig. 2:**
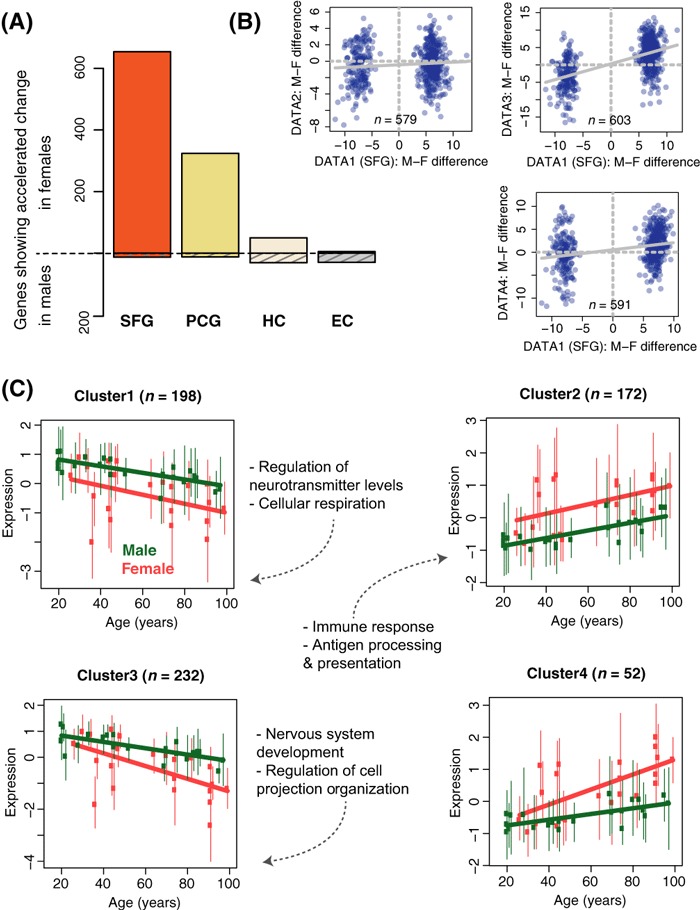
Accelerated aging in the female prefrontal cortex. (A) Heterochrony between the two sexes. The *y*-axis shows the number of genes showing significant heterochrony between the two sexes. The bars above the zero line indicate the number of genes showing accelerated changes in females, those below the zero line indicate genes showing accelerated changes in males. (B) Correlation of sex difference with three additional frontal cortex datasets. *x*-axis: mean sex difference among the 654 female-accelerated genes identified in the Berchtold *et al.* SFG dataset (DATASET1); *y*-axis: sex difference for the same genes in DATASET2 (Colantuoni *et al.*, 2011), DATASET3 (Torkamani *et al.*, 2010). or DATASET4 (Maycox *et al.*, 2009). Sex differences were calculated after normalizing each gene’s expression to mean = 0, standard deviation = 1. The linear regression lines are shown. (C) Expression patterns for 654 female-accelerated genes clustered in four groups; represented as in Fig. 1C. *y*-axis: male (green) and female (red) normalized expression levels; *x*-axis: age in years. Enriched Gene Ontology functions are shown beside each panel (Table S4).

We further sorted genes showing significant heterochrony into two categories: genes whose expression changes significantly faster during female aging, or faster during male aging, relative to the other sex (Fig. 2A; Table S2; Methods). Remarkably, in the SFG, 654 (98%) of the 667 significantly heterochronic genes displayed timing differences in the direction of earlier or faster changes (acceleration) in females, compared to only 13 genes showing acceleration in males. Likewise, 96% of heterochronic genes showed acceleration in females in PCG. This proportion was only 62% in HC and 14% in EC. Together, these results indicate a prominent bias in SFG aging toward accelerated changes in females, in accord with the patterns observed in the clustering analysis. The PCG shows a similar bias but among fewer genes, while the allocortical regions display no such trend.

The bias toward accelerated expression changes in females was unexpected. First, given the longer female life expectancy, one might expect time shifts in the opposite direction, *that is,* acceleration in males. Second, the sheer number of genes showing significant sexual heterochrony in the SFG was surprising, given the modest sexual dimorphism in gene expression identified in earlier studies of the developing and adult brain (Vawter *et al.*, 2004). To rule out any inherent problems with the data or methodology, we first confirmed that population origin and postmortem interval of the subjects did not differ with respect to sex [Wilcoxon test (WT) *P* > 0.1]. Second, we employed two alternative algorithms to test heterochrony, both of which revealed the same bias toward accelerated aging in the female SFG (Methods). Finally, we compared the sex differences identified in the Berchtold *et al.* SFG dataset to those found in three recently published human prefrontal cortex age-series (Table S1, Methods). DATASET2 contained 101 males and 47 females with ages between 20 and 78 years (Colantuoni *et al.*, 2011), DATASET3 contained 19 males and 5 women with ages between 29 and 80 years (Torkamani *et al.*, 2010), and DATASET4 contained nine males and ten females with ages between 38 and 94 years (Maycox *et al.*, 2009). In all three additional datasets, we calculated the mean expression difference between sexes for the 654 genes showing female acceleration in SFG and tested for consistency in the direction of sex differences between datasets (Fig. 2B). This analysis revealed significant correspondence between the Berchtold *et al.* SFG data and each of the additional three datasets [odd’s ratio = 1.9, 13.5, and 2.8, respectively, hypergeometric test (HT) *P* < 0.01 in each test], indicating that accelerated female timing in SFG is a reproducible phenomenon.

### Sexual heterochrony indicates faster aging in females

To gain understanding into the origin and functional consequences of sexual heterochrony, we studied the expression profiles and functional properties of the 654 genes showing accelerated female timing in SFG. We first clustered genes based on their heterochrony patterns identified by DTW-S (Yuan *et al.*, 2011). Namely, we grouped genes based on how the male and female expression time series are shifted with respect to each other (Methods; Fig. S3). We further clustered the genes based on their expression patterns. This resulted in the four clusters shown in Fig. 2C (Table S3). Notably, Clusters 1 and 2 had male and female gene expression trajectories that were largely parallel, indicative of *earlier initiating* changes in females relative to males. Meanwhile, Clusters 3 and 4 showed expression patterns indicative of *faster* changes in females. For simplicity, we refer to both patterns as ‘acceleration.’ Notably, we observed similar sex difference trends among these four clusters using the three additional prefrontal cortex datasets described above (Fig. S4).

We tested these four gene clusters for enrichment in functional categories using the Gene Ontology and KEGG databases (Supporting Information). Specifically, we compared each of the four clusters to all 2490 genes tested for heterochrony in SFG. Three clusters showed significant enrichment in at least one functional process (Bonferroni-corrected HT *P* < 0.05; Table S4): (i) Clusters 1 and 3, which represent genes showing down-regulation with age and lower expression in females, were enriched in neuron-related functions, such as neurotransmitter secretion and neural development. These down-regulated clusters were also significantly enriched in neuron-related genes compared to glia-related genes (Fig. S5). (ii) Cluster 1 was additionally enriched in genes associated with mitochondria and energy production. (iii) Cluster 2, which is up-regulated during aging and shows higher expression in females, was strongly enriched in genes involved in the immune response, particularly autoimmune reactions. Cluster 4 showed no functional enrichment. Meanwhile, applying the same clustering and functional enrichment procedure to genes showing accelerated changes in female PCG revealed only limited enrichment in neural processes in one cluster (Fig. S6, Table S5).

The SFG clusters’ functional enrichment patterns are strongly suggestive, as down-regulation of energy metabolism and neural function-related genes and up-regulation of immune response are major hallmarks of mammalian brain aging (Lee *et al.*, 2000; Lu *et al.*, 2004). This raises the possibility that the observed expression heterochrony is linked to aging-related functional decline in the brain. Accordingly, we confirmed that age-related expression changes among the 654 genes closely paralleled age-related changes in a multi-species prefrontal cortex aging dataset (Somel *et al.*, 2010): genes up-regulated with age and higher expressed in females in the SFG dataset were also up-regulated in both old humans and in old macaques (Fig. S7A–B). Thus, we predict that the 654 genes’ expression patterns represent a trend of earlier or faster age-related functional decline in the female SFG.

### Sexual heterochrony mirrors Alzheimer’s Disease

**Fig. 3:**
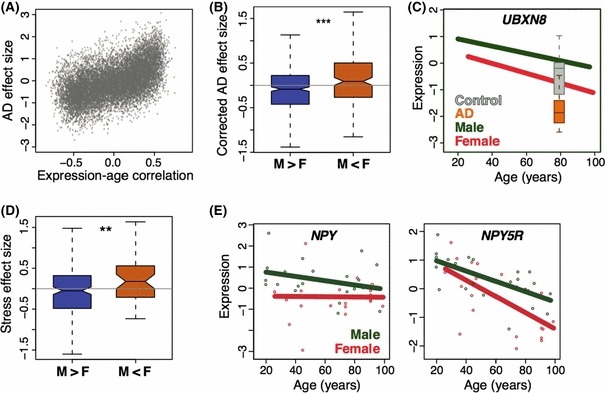
Sex, Alzheimer’s disease, and stress effects on gene expression. (A) Correlation between age-related expression changes and the effect of AD across all expressed genes in SFG. *x*-axis: Pearson correlation coefficient between expression level and age; *y*-axis: effect size between AD and control samples. (B) AD effect among female-accelerated genes. The 654 genes showing female-accelerated aging were separated into two groups: those showing higher expression in females /up-regulation during aging (F > M), or lower expression in females /down-regulation during aging (M > F). *y*-axis: the AD effect size corrected by removing the correlation between the AD and aging effects on expression (Methods). (C) Correlation between AD-related expression effects and sex differences exemplified in the case of *UBXN8* (UBX domain protein 8) expression. *y*-axis: standardized male and female expression levels; *x*-axis: age in years. The expression levels for the AD and control samples were normalized so that the control individuals have the same mean and standard deviation as age-matched individuals in the SFG dataset. (D) The 654 genes showing accelerated female timing separated into two groups as in panel B. The *y*-axis shows the stress effect size on gene expression in male monkey VMPFC, based on (Karssen *et al.*, 2007). (E) Neuropeptide Y and Neuropeptide Y5 receptor expression in the SFG.

If the observed expression patterns are indeed related to functional decline, they might also be associated with aging-related diseases reported to occur at higher frequencies among females, specifically, dementia (Corrada *et al.*, 2008) and AD (Schmidt *et al.*, 2008). To investigate this, we used a dataset of SFG gene expression in individuals with AD (*n* = 23) and age-matched control individuals (*n* = 11) (Liang *et al.*, 2008) (Table S1) and compared age-related change and sexual heterochrony patterns with AD-related expression differences. Interestingly, gene expression changes during normal brain aging were positively correlated with the expression effect of AD (Fig. 3A). We therefore defined a ‘corrected AD effect size’ per gene, a measure of how a gene’s expression is altered under AD, independent of the positive correlation between AD- and aging-related expression changes (Methods). Using this measure, we found that among the 654 accelerated female aging genes, genes up-regulated in females were also significantly up-regulated in AD, and vice versa (Fig. 3B; WT *P* = 0.00013). This result held when using only females among AD-afflicted individuals and controls (*P* < 0.001). This further supports the hypothesis that the accelerated expression changes observed in the female SFG indicate earlier or faster functional decline and a higher propensity to degenerative disease in women (Fig. 3C).

### Accelerated changes may be driven by environmental factors

Why would the female frontal cortex undergo accelerated changes in specific aging-related processes? Functional decline during aging may be promoted by environmental insults, such as psychological stress or trauma (Epel *et al.*, 2004). If women are subject to stronger exposure or display higher vulnerability to stress, this might also be reflected in their SFG transcriptome as a faster aging signal.

If true, we might expect higher expression variation among women, assuming that environmental factors are stochastic and vary in their influence within a population. Indeed, we found that 472 of the 654 accelerated female aging genes (72%) showed significantly higher variance within females than within males (*F*-test, *P* < 0.05), while only one showed the opposite pattern (Table S3). We also noticed that while some females showed accelerated aging trends, others closely followed the male trajectory (Fig. 2C). Using hierarchical clustering and bootstrapping across the 654 genes, we determined that 12 females clustered together with males, while the other ten females (ages varying between 36 and 99 years), formed a distinct group, with expression levels indicating faster age-related changes (Fig. S8). Thus, accelerated SFG aging seems to affect only part of the female population, consistent with the notion that accelerated aging is driven by environmental insults.

One likely culprit is psychological stress, which can have substantial effects on brain structure and function (Brown *et al.*, 2005). In fact, it was shown in male spider monkeys that social stress because of isolation could alter gene expression levels in the frontal cortex (Karssen *et al.*, 2007), notably in the ventromedial prefrontal cortex (VMPFC). When we compared these stress-induced expression changes in the monkey VMPFC and female vs. male differences in human SFG gene expression – despite the dissimilarities between the two studies’ designs – we did find a marginal overlap: across the 654 accelerated female aging genes, those up- and down-regulated in females relative to males also tended to be up- and down-regulated under stress, respectively (Fig. 3D; WT *P* = 0.014).

We also checked possible expression dimorphism in neuropeptide Y (*NPY*) and its receptors. These genes are strongly associated with anxiety and depressive behavior in models organisms and in humans and are down-regulated under stress (Heilig, 2004; Mickey *et al.*, 2011). We found that *NPY*, *NPY1R,* and *NPY5R* show significantly differential expression between males and females (*F*-test *P* < 0.05; Figs 3E and S9), with lower expression in females. Notably, *NPY5R* was also strongly down-regulated in the spider monkey stress experiment. These results hint that psychological stress could be a factor inducing the observed aging-related sex differences in the brain.

## Discussion

Our reanalysis of the Berchtold *et al.* dataset revealed that the vast majority of expression changes in brain aging occur linearly and in the same direction between females and males. Unexpectedly, however, within a considerable proportion (∼5%) of the SFG transcriptome, females reach an ‘aged’ state significantly earlier or faster than males. The reverse pattern was essentially absent.

In addition to the prefrontal cortex, the PCG, part of the somatosensory cortex, also showed significant sexual heterochrony among 2.5% of expressed genes and displayed a clear bias toward accelerated changes in females (Fig. 2A). But heterochronic genes identified in the PCG showed little overlap with heterochronic genes in the SFG (HT *P* > 0.1) and showed weak association with aging-related functional processes, compared to SFG clusters (Table S5). For these reasons, we chose to concentrate our analyses on heterochrony in the SFG.

Notably, the cluster profiles in Fig. 2C implied the existence of two different types of heterochrony: earlier initiating or faster age-related changes in females (represented by clusters 1 & 2, compared to clusters 3 & 4, respectively). The former pattern could also be interpreted as a shifted baseline between sexes, such that adult females, on average, reach the molecular state of older males at a relatively younger age. Earlier initiating vs. faster age-related changes could have distinct causes and mechanisms. However, we did not find differences between gene clusters 1 & 3 or 2 & 4, with respect to functional annotation (data not shown). The datasets used here also do not provide sufficient statistical power to efficiently distinguish between the ‘earlier’ and ‘faster’ models, gene-by-gene. We therefore chose to treat the two types of heterochrony as one and refer to both types simply as ‘acceleration.’

### Is accelerated female prefrontal cortex aging plausible?

Genes showing female acceleration in the SFG have clear associations with brain aging-related processes. Immune reaction pathways, which are up-regulated during normal aging, are up-regulated at higher levels in females compared to same-aged males. Meanwhile, energy metabolism-related genes, which show aging-related decline in expression, are expressed at lower levels in females. We thus interpret the observed sexual expression heterochrony as accelerated aging in the female SFG.

An alternative explanation of the observed sexual heterochrony could be that the observed expression differences are remnants of brain maturation rate differences that arise during adolescence, when female brain maturation is known to proceed slightly faster [reviewed in (Lenroot & Giedd, 2010)]. However, we did not find any comparable indication of sexual heterochrony in a human prefrontal cortex postnatal development dataset (Somel *et al.*, 2009) (Fig. S7C). Thus, the observed sexual heterochrony most likely appears during adulthood, not before.

If the observed expression differences represent faster aging, are the predicted aging rate differences biologically plausible? In other words, given that the study subjects were healthy individuals, is it possible that, as predicted by the DTW-S algorithm (see Fig. S3), certain characteristics of a 50-year-old female brain match that of a centenarian male? In fact, only 5% of the SFG transcriptome shows such stark heterochrony and it is possible that the remainder of the transcriptome partly compensates for the accelerated aging trend in females. Moreover, the observed molecular differences are not direct measures of cognitive differences between sexes. Still, they may reflect differences in propensity for degenerative disorders, like AD.

Importantly, accelerated aging-related changes in females have also been observed in other systems. For example, muscle sympathetic activity, which regulates blood pressure, increases with age more dramatically in women than in men (Narkiewicz *et al.*, 2005). Female reproductive senescence is another example. However, faster female aging in these specific organ systems does not entail faster female aging across all tissues and physiological processes. In the case of brain aging, for instance, we observe strong heterochrony mainly in SFG and at weaker levels in PCG, indicating that heterochrony is not systemic across the brain. Even if the male SFG ages slower than the female SFG, rapid aging in other organ systems, as well as gender- or sex-specific environmental hazards could readily overshadow this trend, culminating in higher male mortality.

### Do accelerated expression changes represent increased AD risk in women?

Another finding that supports a connection between expression heterochrony and aging-related functional decline is the overlap between sex differences and AD effects on the transcriptome. In line with previous reports [*e.g.* (Miller *et al.*, 2008)], we also observe a strong positive correlation between aging-related changes and gene expression changes under AD (Fig. 3A), implying that the AD profile in SFG represents a hyper-aged state. In fact, AD is known to involve the SFG (Covington *et al.*, 2010), and, as mentioned earlier, the condition may also show higher prevalence among elderly women (Andersen *et al.*, 1999; von Strauss *et al.*, 1999; Barnes *et al.*, 2005; Schmidt *et al.*, 2008). Together, these results suggest that the observed expression patterns may represent an increased likelihood of AD development among elderly women.

A challenge to this hypothesis is the lack of strong sexual expression heterochrony in HC and EC, two other regions strongly affected by AD (Raji *et al.*, 2009; Salat *et al.*, 2011). Using the same AD dataset as mentioned earlier, we also find no overlap between sex expression differences identified in the HC and the AD effect in the HC (Fig. S10; WT *P* = 0.23) (Methods). The connection between sexual expression heterochrony and AD thus appears to be unique to the SFG and is not seen in other structures known to show alteration under AD. Nevertheless, the molecular mechanisms of AD progression across different brain regions are not fully known, and it is possible that AD progression is exacerbated by transcriptomic changes unique to SFG. A study of synaptic loss in AD, for example, has found varying trends across regions, with the strongest effect observed in SFG and the weakest in EC (Scheff & Price, 2003). It thus remains conceivable that accelerated expression changes in the female SFG could specifically contribute to AD development in women.

### Can environmental insults drive accelerated female aging and AD?

Intriguingly, the phenomenon of accelerated changes affects about half the female population sampled in these datasets, including women of various ages. Although we lack demographic, clinical, or physiological information on the subjects (except ethnicity), the fact that we observe consistent sex differences across three independent datasets indicates the reproducibility of accelerated female aging in the prefrontal cortex. The expression variation within the female population, in turn, implicates environmental insults in sexual heterochrony. Interestingly, we do find a marginally significant positive correlation between stress-induced expression changes and female–male differences (Fig. 3D–E). This, in turn, fits a number of findings involving sex-specific differential stress response and the role of stress in aging: (i) Stress induces aging-like molecular changes: *for example,* white blood cells of women with higher levels of self-perceived stress have shorter telomeres, a marker of aging (Epel *et al.*, 2004). (ii) The female mammalian brain may be more sensitive to stress: in laboratory rats, acute stress impairs learning in females but not males, an effect depending on the PFC-amygdala pathway (Maeng *et al.*, 2010). Estrogen induces increased dendritic length in PFC neurons that project into the amygdala and increases stress sensitivity (Shansky *et al.*, 2010). Notably, depression also shows higher prevalence among women (Weissman *et al.*, 1996). *(****c****)* Stress-induced changes within the brain can accumulate. For example, stress leads to increased concentrations of hyperpolarized Tau protein and beta-amyloidogenesis in the brain, both of which are molecular changes associated with AD (Devi *et al.*, 2010; Aznar & Knudsen, 2011; Carroll *et al.*, 2011; Sotiropoulos *et al.*, 2011).

These observations and our results together hint that lifelong exposure to higher levels of stress, or higher stress sensitivity, might permanently shift the female SFG transcriptome toward an aged and AD-like state (Joel, 2011). Still, we lack any direct evidence for such a connection. Notably, factors such as reproductive history or past trauma could also influence the SFG transcriptome and induce aging-like changes.

Finally, we note that the ten females showing accelerated aging-like expression patterns in the SFG (Fig. S8) also tended to show divergent expression patterns in other brain regions (Fig. S11), although to lesser extent. This suggests that the causal factor of divergent expression in these individuals, possibly stress, may affect multiple brain regions simultaneously, although it is detected as a faster aging signal only in SFG. This discrepancy may be due to differences in aging dynamics among the brain regions.

## Conclusion

Here, we have identified a conspicuous trend toward faster female aging in the prefrontal cortex using transcriptome analysis. It remains to be shown which endocrinological, psychological, and medical conditions give rise to faster SFG aging rates. It is also unclear whether the observed sex differences reflect temporary differences among individuals, or an accumulating, irreversible molecular load. Future work using larger and better-annotated human postmortem datasets, and brain transcriptome analyses of mammalian stress models, could help identify any causal relationships between sex differences in brain aging, the effects of stress, and disease susceptibility.

## Methods

### Dataset preprocessing

We conducted heterochrony analysis with the gene expression age-series dataset reported in (Berchtold *et al.*, 2008), available at the NCBI GEO database (http://www.ncbi.nlm.nih.gov/geo/; GEO ID GSE11882), and which we refer as DATASET1. This is based on Affymetrix HG-U133Plus2.0 microarrays where gene expression was measured in healthy males and females, ∼20 per sex, across four brain regions. CEL files were processed in the R environment; expression levels per gene were summarized, log-transformed, and quantile normalized using the R ‘affy’ library ‘rma’ function. Probe sets were defined based on Ensembl genes (Dai *et al.*, 2005) (ensembl version 61). We excluded 13 samples with potential sex mislabeling or technical problems: (i) we checked sex identity by *XIST* and Y-chromosome-linked gene expression, which revealed atypical expression (too high or too low) in ten samples (accession IDs: GSM300213, GSM300250, GSM318840, GSM300288, GSM300287, GSM300326, GSM300212, GSM300255, GSM300192, GSM300300; four females & six males). (b) We checked the overall variation among individuals in each region using principle components analysis (PCA) and k-means clustering, using the R ‘prcomp’ and ‘kmeans’ functions. This revealed three samples as potential outliers (accession IDs: GSM300198, GSM300301, GSM300196; two females & one male). Specifically, in k-means clustering, we found that for 400–600 genes, these samples had mean expression level >3 standard deviations distant from the average mean, whereas all other samples were within one standard deviation. For the analyses presented in the main text, we preprocessed CEL files for each brain region separately, and without including the outliers/misidentified individuals.

### Tests for age and differential expression effects

For testing the effect of age on expression levels per gene, we used a family of polynomial regression-based tests, and for testing differential expression between two series, an analysis of covariance-based test. Both are based on the adjusted *r*^2^ criterion and are described in (Somel *et al.*, 2009). Importantly, our approach differs from the original analysis, which had sorted individuals into four distinct age-bins and had identified age-related genes by comparing each group to the consecutive one (Berchtold *et al.*, 2008). Relative to this approach, modeling the data using regression models can increase statistical power to identify gradual changes across lifespan.

Consequently, we selected 2490 genes in SFG, 2102 genes in PCG, 584 genes in HC, and 186 genes in EC as the test gene set for applying DTW-S, satisfying the following criteria: (i) significant expression change with age, (ii) significant expression difference between males and females, and (iii) significant positive correlation (co-directional change) between male and female expression profiles (Data S1).

### Heterochrony analyses with DTW-S

We used the DTW-S algorithm to analyze heterochrony (Yuan *et al.*, 2011). Compared to similar warping algorithms, DTW-S has a number of advantages, such as relaxing the end-matching requirement, estimation of shifts per time point per gene, the introduction of a significance test for the identified heterochrony patterns based on simulation, and fast implementation. Using the gene sets defined above, for each gene, we aligned female expression trajectories to male trajectories, and vice versa. In each case, we estimated the time-shift (heterochrony) between the aligned trajectories and conducted simulations to estimate the significance of the shift (Yuan *et al.*, 2011). We considered genes as ‘significantly heterochronic’ if they showed shift at *P* < 0.05 in both alignments (Fig. S12, Table S2).

### Additional human brain aging datasets

We used three additional datasets to confirm our results. DATASET2 (Colantuoni *et al.*, 2011) is based on Illumina BeadChips and contains 158 males and 73 females with ages between 0 and 78 years (GEO ID: GSE30272). DATASET3 (Torkamani *et al.*, 2010) (GEO ID: GSE21138) contains 19 males and five females with ages between 29 and 80 years. DATASET4 (Maycox *et al.*, 2009) (GEO ID: GSE17612) contains nine males and ten females with ages between 38 and 94 years; the latter two are based on Affymetrix HG-U133Plus2.0 arrays (Table S1). For quality control and preprocessing information, see Data S1.

### AD and stress datasets

To gain functional insight into the identified sexual heterochrony, we used two datasets, one studying the effects of Alzheimer’s disease on the human brain transcriptome (Liang *et al.*, 2008) using Affymetrix HG-U133Plus2.0 arrays, the other, the effects of social stress on the spider monkey brain transcriptome (Karssen *et al.*, 2007) using Affymetrix HG-U133A2.0 arrays. The SFG subset of the AD expression dataset (GEO ID: GSE5281) contained *n* = 23 AD-afflicted individuals (age range, 68–95 years) and *n* = 11 age-matched controls (age range, 63–102 years). Both groups contained ∼60% males. The HC subset of the AD expression dataset contained *n* = 10 AD-afflicted individuals and *n* = 13 age-matched controls. We calculated the Alzheimer’s disease effect size for each gene using the Cohen’s D measure. Given the correlation between these values and the expression-age Pearson correlation coefficient across genes (Fig. 3A), we calculated residuals from a linear regression model between AD effect size vs. the age-expression correlation coefficient across genes, this measure we treated as a ‘corrected effect size’. Repeating the comparison using only females in the AD dataset revealed the same significant relationship between SFG sex differences and AD, as observed using all subjects (*P* < 0.0001), while using only males revealed no signal (*P* > 0.1).

The VMPFC subset of the spider monkey dataset was downloaded from http://www.pritzkerneuropsych.org/?page_id=400 (the RMA dataset preprocessed by the authors). This contained *n* = 9 stressed (at juvenile or adult stage) and *n* = 3 control (not stressed at either stage) individuals.

### Additional heterochrony and functional analysis

We applied the original DTW algorithm (Aach & Church, 2001) and an alternative algorithm based on nonlinear least squares (Somel *et al.*, 2009), on all the genes tested for heterochrony in SFG. Both methods yielded a clear excess of genes showing female acceleration (92% and 96%, respectively). Details of functional analyses are provided in Data S1.

## Supporting Information

**Fig. S1** Age-distribution for the samples used in this study.

**Fig. S2** Sexually dimorphic expression patterns in PCG, HC and EC.

**Fig. S3** Two main time-shift patterns among the SFG female-accelerated genes.

**Fig. S4** Expression patterns of SFG female-accelerated gene clusters in three additional datasets.

**Fig. S5** Cell type over-representation among the SFG female-accelerated gene clusters.

**Fig. S6** Expression patterns for 324 female-accelerated genes in PCG.

**Fig. S7** Expression patterns of SFG female-accelerated gene clusters in development and aging.

**Fig. S8** Clustering of individuals reveals subgrouping within females.

**Fig. S9** Sexual dimorphism in *NPY* and *NPYR* expression.

**Fig. S10** A.D. effect size for sexually dimorphic genes in HC.

**Fig. S11** Male-female expression distances in faster aging and slower aging females.

**Fig. S12** Number of genes showing significant sexual heterochrony in four brain regions.

**Table S1** Datasets used in the study.

**Table S2** Sexual heterochrony across the four brain regions.

**Table S3** Gene names for the four female-accelerated clusters in the superior frontal gyrus (SFG).

**Table S4** Biological processes over-represented among female-accelerated genes in the superior frontal gyrus (SFG).

**Table S5** Biological processes over-represented among female-accelerated genes in the postcentral gyrus (PCG).

**Data S1** Supporting materials and methods.

As a service to our authors and readers, this journal provides supporting information supplied by the authors. Such materials are peer-reviewed and may be re-organized for online delivery, but are not copy-edited or typeset. Technical support issues arising from supporting information (other than missing files) should be addressed to the authors.
