# Supplementary material for: Accelerated aging-related transcriptome changes in the female prefrontal cortex
Source: Aging Cell. 2012 Oct;11(5):894–901. doi: 10.1111/j.1474-9726.2012.00859.x (PMC3470704; doi:10.1111/j.1474-9726.2012.00859.x)
Supplement: Supplementary file 1 [file acel0011-0894-SD1.pdf]

Supporting Information

Accelerated aging-related transcriptome changes in the female prefrontal cortex

Supporting tables

Table S1. Datasets used in the study. 2
Table S2. Sexual heterochrony across the four brain regions. 3
Table S3. Gene names for the four female-accelerated clusters in the superior frontal gyrus (SFG). 4
Table S4. Biological processes over-represented among female-accelerated genes in the superior frontal gyrus (SFG). 9
Table S5. Biological processes over-represented among female-accelerated genes in the postcentral gyrus (PCG). 11

Supporting figures

Figure S1. Age-distribution for the samples used in this study. 12
Figure S2. Sexually dimorphic expression patterns in PCG, HC and EC. 13
Figure S3. Two main time-shift patterns among the SFG female-accelerated genes. 15
Figure S4. Expression patterns of SFG female-accelerated gene clusters in three additional datasets. 16
Figure S5. Cell type over-representation among the SFG female-accelerated gene clusters. 18
Figure S6. Expression patterns for 324 female-accelerated genes in PCG. 19
Figure S7. Expression patterns of SFG female-accelerated gene clusters in development and aging. 20
Figure S8. Clustering of individuals reveals subgrouping within females. 21
Figure S9. Sexual dimorphism in *NPY* and *NPYR* expression. 22
Figure S10. A.D. effect size for sexually dimorphic genes in HC. 23
Figure S11. Male-female expression distances in faster aging and slower aging females. 24
Figure S12. Number of genes showing significant sexual heterochrony in four brain regions. 25

Supporting notes

DATASET1 quality control. 26
Spline interpolation. 26
Linear vs. non-linear changes 26
Positive correlation between sexes. 27
False discovery rate estimation. 27
Clustering. 27
DTW-S application. 27
Additional human brain aging datasets. 28
Functional analysis 29

Supporting tables

Table S1. Datasets used in the study.

Dataset symbol	Species	Brain region	Description of samples*	Microarray type	Reference; GEO id or link
DATASET1	Human	SFG	22 healthy males and 22 healthy females (age range: 20-99 years)	Affymetrix HG-U133Plus 2.0	Berchtold et al. 2008; GSE11882
		PCG	21 healthy males and 18 healthy females (age range: 20-99 years)		
		HC	21 healthy males and 20 healthy females (age range: 20-99 years)		
		EC	20 healthy males and 16 healthy females (age range: 20-99 years)		
DATASET2	Human	PFC	158 healthy males and 73 healthy females (age range: 0-78 years)	Illumina BeadChip	Colantuoni et al. 2011; GSE30272
DATASET3	Human	PFC	19 healthy males and 5 healthy females (age range: 29-80 years)	Affymetrix HG-U133Plus 2.0	Torkamani et al. 2010; GSE21138
DATASET4	Human	PFC	9 healthy males and 10 healthy females (age range: 38-94 years)	Affymetrix HG-U133Plus 2.0	Maycox et al. 2009; GSE17612
AD	Human	SFG	23 AD-afflicted individuals (age range: 68-95 years) and 11 age-matched controls (age range: 63-102 years)	Affymetrix HG-U133Plus 2.0	Liang et al. 2008; GSE5281
		HC	10 AD-afflicted individuals (age range: 70-88 years) and 13 age-matched controls (age range: 63-102 years).		
Stress	Spider monkey	VMPFC	9 stressed (at juvenile or adult stage) and 3 control (not stressed at either stage) individuals	Affymetrix HG-U133A 2.0	Karssen et al. 2007; http://www.pritzkerneuropsych.org/?page_id=400

*Samples after removing potential outliers and sex mislabeled individuals. See Methods and Supporting Notes for details.

Table S2. Sexual heterochrony across the four brain regions.

The table describes the number of genes in DATASET1 showing significant age-related expression changes in either male or female (A.R.); showing both significant age-related changes and dimorphic expression changes between two sexes (A.R. and D.E.); showing significant age-related changes and parallel expression patterns between two sexes (A.R. and P.E.); showing significant age-related expression changes, parallel expression patterns and dimorphic expression changes between two sexes (A.R. and P.E. and D.E.); showing significant time shift; showing significant positive time shift (see below); showing significant negative time shift; showing no direction in terms of time shift. There are 13910 expressed genes in total. M to F: aligning male expression trajectories to female expression trajectories; here positive time shift indicates faster changes in males. F to M: aligning female expression trajectories to male expression trajectories; here positive time shift indicates faster changes in females. The red marked cells give the number of genes showing female accelerated aging. FPR: false positive rate.

	A.R.	A.R. and D.E.	A.R. and P.E.	A.R. and P.E. and D.E.	Significant time shift (FPR)	Positive shift[a]	Negative shift[b]	None
SFG (M to F)	7745	2850	6668	2490	930 (12%)	8	897	15
SFG (F to M)	7745	2850	6668	2490	1848 (9.5%)	1824	19	5
PCG (M to F)	6709	2545	5477	2102	473 (25%)	14	442	17
PCG (F to M)	6709	2545	5477	2102	1009 (10%)	974	20	15
HC (M to F)	5295	927	4064	584	176 (11.7%)	57	93	26
HC (F to M)	6709	2545	5477	2102	156 (11%)	113	43	0
EC (M to F)	3743	475	2626	186	146 (10%)	49	46	51
EC (F to M)	3743	475	2626	186	41 (17%)	6	34	1

[a]Positive shift: Faster changes in males in M to F alignment, faster changes in females in F to M alignment.

[b]Negative shift: Faster changes in females in M to F alignment, faster changes in males in F to M alignment.

Table S3. Gene names for the four female-accelerated clusters in the superior frontal gyrus (SFG).

The table lists the gene names (HGNC symbols) and Ensemble gene IDs for four clusters (C1-4) of female-accelerated genes. Genes with significantly higher female expression variance are marked in orange, one gene with significantly higher male expression variance is marked in green and genes not showing expression variance difference are marked in blue.

Female-accelerated cluster	HGNC gene symbols	Ensembl gene ID
C1 (n=198)	GAD1, MEAF6, NEUROD6, SEH1L, PWP1, TCEAL7, NGFRAP1, CSPG5, ATP5A1, SMCR7L, ZNF711, NXPH1, BHLHB9, SLC25A32, STAM, SUMO3, SYN1, AMZ2P1, NF1, CUL2, MRPL15, GTF3C4, ATP8A2, RRAGA, COX5A, ZNF233, GABBR1, FOXG1, TMEM66, PCDH10, ATP6V1B2, TRMT12, ZNF238, ADSS, IMP3, NRG3, TTPAL, SDHB, SNCA, CCT2, CLASP2, TM2D3, TTLL1, FBLL1, MBTPS2, TMEM19, TPPP, UCHL1, C17orf69, APOO, TFDP1, FOXJ3, PITPNB, CADPS, TUBA1B, CLTC, PEBP1, MAGED1, SEC61G, PTRH2, SPIN1, ARIH1, HPRT1, SLC30A9, TTC3, MYADM, SYT1, FARS2, C1orf128, HTR2C, SNAPC5, ATP6V1A, RAB3GAP1, GLS, SLC25A40, ZCCHC12, RAB14, RBM18, TMEFF2, NDUFAB1, PPIL1, PGRMC1, PSMG1, CMAS, ST8SIA1, KIAA1279, PARM1, NDUFV1, DCLK1, PJA1, STMN1, FEM1B, MRPS9, ERC2, HIVEP1, CHCHD1, ING4, GDAP1, TBC1D7, CHGB, COX7A2, RANBP9, MDH1, ANKRD32, BCAS2, SRP19, TADA1, GUCY1B3, CTNND2, PDHB, PPP6C, SNAP25, LDOC1, NECAP1, FAM98B, C6orf120, TBCC,	ENSG00000014824, ENSG00000085415, ENSG00000090316, ENSG00000097021, ENSG00000106723, ENSG00000112379, ENSG00000117632, ENSG00000125484, ENSG00000132432, ENSG00000133872, ENSG00000143363, ENSG00000147180, ENSG00000147246, ENSG00000152382, ENSG00000163875, ENSG00000164600, ENSG00000166233, ENSG00000166681, ENSG00000169116, ENSG00000169862, ENSG00000170846, ENSG00000171262, ENSG00000174405, ENSG00000174460, ENSG00000179456, ENSG00000179820, ENSG00000180957, ENSG00000181191, ENSG00000184277, ENSG00000205060, ENSG00000250702, ENSG00000004779, ENSG00000008056, ENSG00000010017, ENSG00000012174, ENSG00000014641, ENSG00000035687, ENSG00000057757, ENSG00000061918, ENSG00000067715, ENSG00000070761, ENSG00000075303, ENSG00000075790, ENSG00000089199, ENSG00000089220, ENSG00000089818, ENSG00000095002, ENSG00000095951, ENSG00000100271, ENSG00000100335, ENSG00000101856, ENSG00000104381, ENSG00000108094, ENSG00000111653, ENSG00000111726, ENSG00000111728, ENSG00000112695, ENSG00000114573, ENSG00000114646, ENSG00000114686, ENSG00000115365, ENSG00000115419, ENSG00000115839, ENSG00000116752, ENSG00000117118, ENSG00000119396, ENSG00000119414, ENSG00000119446, ENSG00000122584, ENSG00000122873, ENSG00000123416, ENSG00000124120, ENSG00000124659, ENSG00000128683, ENSG00000129084, ENSG00000131779, ENSG00000132639, ENSG00000132932, ENSG00000133083, ENSG00000133302, ENSG00000133997, ENSG00000135324, ENSG00000135624, ENSG00000135972, ENSG00000136045, ENSG00000136738, ENSG00000137168, ENSG00000137547, ENSG00000138650, ENSG00000139291, ENSG00000141367, ENSG00000141378, ENSG00000144120, ENSG00000144339,

	KIAA1244, LANCL1, ANKLE2, PEX11B, MAEA, TOMM70A, PSMA1, NDUFV3, MRAP2, RXFP1, CISD1, ACOT7, GSTA4, PRUNE, NRSN1, SLC35B4, ATP6V0C, LIG4, BPGM, BCAP29, MSH2, TOMM20, MED6, C16orf80, TMEM177, RNASEK, MRPL3, CCT7, SCG2, ISLR2, KIAA1715, DLX6-AS, KCNF1, CHN2, BMPER, NUAK1, UBXN8, GAD2, EXOSC3, FARSA, SEC22C, CCNY, CPNE6, ENDOG, ZNF239, TTC9B, PCDH11Y, TMSB4Y, SYT11, NMU, KIF2A, CELF6, TBC1D10B, WDR44, SST, ANKRD28, NNAT, NCAM2, PTBP2, TOX3, RAB3B, GRIK1, EPHA3, DNAJC30, MFI2, TM9SF2, CCDC136, PCDHB14, MFSD3, TMEM87B, C1orf174, BIVM, FJX1, KIAA0232, ANKRD6, MBLAC1, TMEM5	ENSG00000145335, ENSG00000145979, ENSG00000145982, ENSG00000147416, ENSG00000152234, ENSG00000152954, ENSG00000153037, ENSG00000154174, ENSG00000154277, ENSG00000155876, ENSG00000159915, ENSG00000160194, ENSG00000163539, ENSG00000163618, ENSG00000164933, ENSG00000165704, ENSG00000166226, ENSG00000166503, ENSG00000167159, ENSG00000167792, ENSG00000168291, ENSG00000169018, ENSG00000170899, ENSG00000171368, ENSG00000171509, ENSG00000171951, ENSG00000172331, ENSG00000172586, ENSG00000173726, ENSG00000174446, ENSG00000176165, ENSG00000176915, ENSG00000177971, ENSG00000178741, ENSG00000179222, ENSG00000182195, ENSG00000182670, ENSG00000182916, ENSG00000183527, ENSG00000183665, ENSG00000184831, ENSG00000184900, ENSG00000185127, ENSG00000185737, ENSG00000185883, ENSG00000187672, ENSG00000188573, ENSG00000196712, ENSG00000198176, ENSG00000198815, ENSG00000198908, ENSG00000198954, ENSG00000204681, ENSG00000214174, ENSG00000219200, ENSG00000250056, ENSG00000167178, ENSG00000044524, ENSG00000053438, ENSG00000093183, ENSG00000099715, ENSG00000104691, ENSG00000107371, ENSG00000108100, ENSG00000109255, ENSG00000120327, ENSG00000128596, ENSG00000131725, ENSG00000132718, ENSG00000134897, ENSG00000135299, ENSG00000140488, ENSG00000144320, ENSG00000153214, ENSG00000162975, ENSG00000163975, ENSG00000164619, ENSG00000167700, ENSG00000170871, ENSG00000174521, ENSG00000179431, ENSG00000196793, ENSG00000198912, ENSG00000206560, ENSG00000214309, ENSG00000231764, ENSG00000068796, ENSG00000074590, ENSG00000100884, ENSG00000103460, ENSG00000106069, ENSG00000117569, ENSG00000118600, ENSG00000125304, ENSG00000136750, ENSG00000154620, ENSG00000154654, ENSG00000157005, ENSG00000167136, ENSG00000169213, ENSG00000169221, ENSG00000171189, ENSG00000176410, ENSG00000179115
**C2 (n=172)**	CRNDE, SIN3B, CSF3R, USP4, MS4A14, C11orf63, DNAJC4, SQSTM1, NCRNA00183, SLC7A6OS, LCNL1, NTS, PCNT, SLC2A4RG, ZNF844, BCL2A1, HLA-E, TGS1, CEL, RPS21, SLC25A37, PVRL2, PLIN2, OSMR, HLA-F, PRKD2, RNASE2, SNX33, ZRSR2, SAMD4B, RPL37, C7, RAC2, NQO1, IRF3, RPS19, TRIM38, DOK3, SAT1, VSIG10, ELTD1, ZNF600, MDFIC,	ENSG00000065911, ENSG00000103061, ENSG00000105287, ENSG00000105372, ENSG00000110011, ENSG00000116786, ENSG00000125520, ENSG00000130202, ENSG00000138794, ENSG00000158710, ENSG00000166523, ENSG00000169249, ENSG00000171115, ENSG00000176834, ENSG00000177666, ENSG00000179134, ENSG00000183888, ENSG00000189190, ENSG00000204592, ENSG00000221963, ENSG00000018280, ENSG00000026950, ENSG00000047617, ENSG00000064012, ENSG00000081320, ENSG00000083444, ENSG00000085276, ENSG00000091879, ENSG00000099875, ENSG00000100292, ENSG00000109944, ENSG00000110852, ENSG00000112312, ENSG00000112343, ENSG00000112936, ENSG00000114316,

	CXCL1, GBP2, MR1, CDKN1A, STK17B, CASP6, PNPLA2, TAGLN2, KDM6B, MECOM, ANGPT2, ANO2, CASP8, BTN3A1, GIMAP8, GIMAP4, CDK2, PER1, NAAA, SEC14L1, SH2B2, CLEC4E, TNFAIP3, PLEKHH2, CLEC2B, HMOX1, APOL6, MKNK2, SLC11A1, GMNN, IL6R, PLOD1, CCNT1, DNPEP, MYO1F, MTHFD2, PLEKHM2, SLC25A34, C1orf64, NCRNA00282, GNB2L1, EYA3, EPB41, PSMB8, SSFA2, TAP1, ATL3, HELLS, C5orf56, BTK, RAB27A, MLLT6, FADS3, KDM6A, TNFRSF10B, KHNYN, TGFBR2, EMR2, SLCO4A1, ANXA3, AKAP13, HLA-C, SLC1A5, HLA-B, HLA-A, CLIC2, ZNF502, SNRPA, PACSIN2, RPS11, RCN3, PARP16, SGMS2, DUSP18, SIGLEC10, HIST1H1C, PAPSS2, FKBP5, SAMHD1, TMEM106A, ATP7B, SIX4, SQRDL, COG7, PXN, PRKCH, RPL31, STEAP4, IL1R1, LAMC1, TGM2, USP3, CYYR1, PLA1A, CALCOCO2, ARHGAP30, ZBTB16, GRK4, IL10RB, TAGLN, LMO2, CP, NLRC5, FAM179A, TM4SF1, C10orf11, LCP1, VASH2, NFKBIZ, EPAS1, PLBD1, GNG11, TMEM43, SASH3, S100A14, CMAH, PARP12, SP110, SP140L	ENSG00000118503, ENSG00000119535, ENSG00000123374, ENSG00000123992, ENSG00000124762, ENSG00000126456, ENSG00000127511, ENSG00000128340, ENSG00000129315, ENSG00000129657, ENSG00000130066, ENSG00000132510, ENSG00000133574, ENSG00000133636, ENSG00000135272, ENSG00000137574, ENSG00000138744, ENSG00000140379, ENSG00000142347, ENSG00000145592, ENSG00000145623, ENSG00000146094, ENSG00000147454, ENSG00000147872, ENSG00000152527, ENSG00000153029, ENSG00000160299, ENSG00000160712, ENSG00000160999, ENSG00000161011, ENSG00000162461, ENSG00000162618, ENSG00000162645, ENSG00000163739, ENSG00000166928, ENSG00000169385, ENSG00000170835, ENSG00000171858, ENSG00000173548, ENSG00000179094, ENSG00000181019, ENSG00000204642, ENSG00000214402, ENSG00000223547, ENSG00000225470, ENSG00000229348, ENSG00000232611, ENSG00000245694, ENSG00000247217, ENSG00000253661, ENSG00000010671, ENSG00000027075, ENSG00000047457, ENSG00000059378, ENSG00000077312, ENSG00000089159, ENSG00000096060, ENSG00000100266, ENSG00000100441, ENSG00000100625, ENSG00000101187, ENSG00000109906, ENSG00000116016, ENSG00000119969, ENSG00000120889, ENSG00000125388, ENSG00000135363, ENSG00000135899, ENSG00000136167, ENSG00000136436, ENSG00000137767, ENSG00000138434, ENSG00000138617, ENSG00000140455, ENSG00000142534, ENSG00000142552, ENSG00000148655, ENSG00000159023, ENSG00000163513, ENSG00000164023, ENSG00000166265, ENSG00000168405, ENSG00000168434, ENSG00000170876, ENSG00000184743, ENSG00000185404, ENSG00000196653, ENSG00000197536, ENSG00000198682, ENSG00000198959, ENSG00000204264, ENSG00000204525, ENSG00000204628, ENSG00000221968, ENSG00000234707, ENSG00000243754, ENSG00000246013, ENSG00000248072, ENSG00000249648, ENSG00000069974, ENSG00000071082, ENSG00000101347, ENSG00000105281, ENSG00000108292, ENSG00000115594, ENSG00000121316, ENSG00000122122, ENSG00000123191, ENSG00000127507, ENSG00000127920, ENSG00000127954, ENSG00000135862, ENSG00000138772, ENSG00000140853, ENSG00000142512, ENSG00000143494, ENSG00000144802, ENSG00000144837, ENSG00000147050, ENSG00000149591, ENSG00000155962, ENSG00000158161, ENSG00000167065, ENSG00000168394, ENSG00000169908, ENSG00000170776, ENSG00000184988, ENSG00000186517, ENSG00000187837, ENSG00000189334, ENSG00000189350, ENSG00000206503, ENSG00000234745, ENSG00000234771, ENSG00000243646, ENSG00000251209
**C3 (n=232)**	UBR3, SCN2A, DLX1, CDK5R1,	ENSG00000005020, ENSG00000017260, ENSG00000022355, ENSG00000025796,

	ANKMY2, SNX10, PDE1A, SKAP2, CAMKV, ZC3H15, FAM49A, CDK17, RDH14, NICN1, RTN3, SCN3B, VAPA, RBP4, DMXL2, TMOD2, BEX2, OXR1, B4GALT6, BSCL2, YWHAZ, TCEAL1, SYT4, RSL24D1, NCALD, KIF3A, SVOP, PNMA1, RAB2A, ARMCX2, BEX5, SCOC, ACSL6, BRMS1L, SEC23A, TMEM155, UBE2V2, COPS4, NEFL, AP3M2, ATP2B1, UBE2N, CCK, BTBD1, GABRA1, GABRG2, MAL2, HCN1, CAND1, GRIA1, YWHAB, RAB39B, RCN2, DCK, PLK2, USP46, WDR7, YIPF5, MANEAL, TUBB2A, OXCT1, TRHDE, PTPRA, RGS17, CRYM, ATOH7, FAM179B, EAF1, ITFG1, DENR, RTN1, ST6GALNAC5, RNF111, UQCRC2, GDA, CDC42, WSB2, C11orf87, RAB11A, NSF, UBE3A, CNIH3, HSPA12A, PRKACB, LCLAT1, GLOD4, FAM174A, GABRB3, GABRA5, STAMBPL1, RTN4, KPNA4, UBE2D2, MFSD6, KIF3B, LRRC8B, KCNJ6, DUSP28, TMEM181, MOAP1, NELL1, DIRAS2, SLC39A10, FAM19A1, CALB1, C3orf59, SMAP1, PRKCB, TCP1, GAP43, ATL1, DLK2, SEC63, SERPINI1, HIAT1, EXTL2, RC3H2, ATP9A, EIF4E3, PRMT6, EPS15, C13orf36, FAM102B, TMEM132B, SLC25A46, ROBO2, MZT1, KLHDC5, NKIRAS1, GPR137C, RAN, SDAD1, SUMO1, PA2G4, PARG, C14orf129, YWHAH, FGFR1OP, STMN2, DNAJC6, SGIP1, REEP1, KBTBD7, SLC4A1AP, REEP5, SYN2, TBL1XR1, LRRC40, NEGR1, CAPRIN1, C10orf32, GLRB, C3orf26, CAMSAP1L1, VPS35, SNAP91, NAA30, ACTR3B, C6orf115, SNX14, PREPL, RG9MTD1, TSGA14, TSPYL5, MAGEH1, SSTR2, TMEM14A,	ENSG00000047932, ENSG00000050030, ENSG00000054793, ENSG00000059758, ENSG00000064726, ENSG00000065548, ENSG00000065609, ENSG00000066557, ENSG00000068615, ENSG00000070831, ENSG00000070961, ENSG00000072657, ENSG00000073969, ENSG00000083720, ENSG00000086300, ENSG00000087448, ENSG00000091129, ENSG00000091157, ENSG00000091844, ENSG00000091972, ENSG00000096092, ENSG00000100744, ENSG00000100916, ENSG00000100934, ENSG00000101350, ENSG00000101558, ENSG00000103316, ENSG00000104093, ENSG00000104327, ENSG00000104388, ENSG00000104490, ENSG00000104725, ENSG00000106477, ENSG00000109189, ENSG00000109738, ENSG00000110768, ENSG00000111110, ENSG00000111530, ENSG00000114062, ENSG00000115252, ENSG00000115310, ENSG00000116030, ENSG00000116675, ENSG00000117069, ENSG00000117600, ENSG00000117906, ENSG00000118473, ENSG00000119125, ENSG00000120438, ENSG00000120696, ENSG00000123545, ENSG00000125629, ENSG00000127824, ENSG00000128245, ENSG00000129625, ENSG00000129636, ENSG00000131437, ENSG00000131508, ENSG00000132341, ENSG00000132670, ENSG00000132872, ENSG00000133019, ENSG00000133134, ENSG00000133318, ENSG00000133627, ENSG00000134375, ENSG00000135002, ENSG00000135317, ENSG00000136143, ENSG00000136531, ENSG00000137267, ENSG00000137876, ENSG00000138134, ENSG00000138207, ENSG00000138663, ENSG00000139364, ENSG00000139726, ENSG00000139970, ENSG00000139977, ENSG00000142875, ENSG00000143786, ENSG00000144355, ENSG00000144357, ENSG00000144567, ENSG00000144597, ENSG00000145029, ENSG00000145632, ENSG00000145817, ENSG00000146386, ENSG00000146433, ENSG00000147676, ENSG00000151690, ENSG00000153130, ENSG00000153956, ENSG00000155511, ENSG00000156136, ENSG00000156875, ENSG00000157152, ENSG00000157450, ENSG00000157542, ENSG00000162636, ENSG00000163444, ENSG00000163536, ENSG00000163781, ENSG00000163798, ENSG00000164076, ENSG00000164112, ENSG00000164398, ENSG00000164588, ENSG00000164830, ENSG00000164924, ENSG00000165023, ENSG00000165434, ENSG00000165868, ENSG00000165943, ENSG00000165973, ENSG00000166111, ENSG00000166206, ENSG00000166257, ENSG00000166275, ENSG00000166352, ENSG00000166501, ENSG00000166913, ENSG00000167699, ENSG00000169139, ENSG00000170091, ENSG00000170515, ENSG00000171462, ENSG00000172020, ENSG00000172260, ENSG00000172465, ENSG00000172954, ENSG00000174132, ENSG00000175497, ENSG00000176749, ENSG00000176871, ENSG00000176903, ENSG00000177565, ENSG00000177889, ENSG00000179774,

	GOPC, SUCLA2, TIMM17A, MLLT11, DPP10, C11orf74, INSIG2, KIAA2022, TMEM183A, C17orf100, ATP2C1, NRCAM, FAM134A, TAGLN3, CD200, CACNA2D1, PGM2L1, TUBA4A, GTF2H1, TOPBP1, FAM19A2, CHRM3, PPM1H, NDUFAF4, GRIN3A, SSTR1, SERINC3, PTPRR, ARPP21, ASPHD2, VIP, CELF5, NPY5R, ARHGAP20, PRKCG, MAGI1, PPP4R4, HS2ST1, LMO4, LHX6, PPP2R5E, LRRTM1, EFNB3, EPHA5, BCL11A, STX1A, GNAO1, C4orf44, LARGE, PAK7, STYK1, MMD, MAP3K12, TRIM46, PID1	ENSG00000180440, ENSG00000180543, ENSG00000180611, ENSG00000180616, ENSG00000180998, ENSG00000183662, ENSG00000184220, ENSG00000184515, ENSG00000184867, ENSG00000185008, ENSG00000185090, ENSG00000185742, ENSG00000186297, ENSG00000186432, ENSG00000187601, ENSG00000196950, ENSG00000197147, ENSG00000197872, ENSG00000197885, ENSG00000198513, ENSG00000198673, ENSG00000198718, ENSG00000198890, ENSG00000204899, ENSG00000212734, ENSG00000227345, ENSG00000233137, ENSG00000233705, ENSG00000240857, ENSG00000056586, ENSG00000069329, ENSG00000070718, ENSG00000085832, ENSG00000103769, ENSG00000104435, ENSG00000106524, ENSG00000112305, ENSG00000113327, ENSG00000118200, ENSG00000118276, ENSG00000128872, ENSG00000135387, ENSG00000138078, ENSG00000140740, ENSG00000144834, ENSG00000155961, ENSG00000162694, ENSG00000163412, ENSG00000164209, ENSG00000168000, ENSG00000174173, ENSG00000187094, ENSG00000188542, ENSG00000198301, ENSG00000213066, ENSG00000213190, ENSG00000060140, ENSG00000087258, ENSG00000101349, ENSG00000106089, ENSG00000106852, ENSG00000108947, ENSG00000108960, ENSG00000119698, ENSG00000119866, ENSG00000126583, ENSG00000128203, ENSG00000132824, ENSG00000133424, ENSG00000137727, ENSG00000139625, ENSG00000139874, ENSG00000143013, ENSG00000145242, ENSG00000146469, ENSG00000151276, ENSG00000153233, ENSG00000153823, ENSG00000153936, ENSG00000154001, ENSG00000161082, ENSG00000162951, ENSG00000163462, ENSG00000164129, ENSG00000172995, ENSG00000188981, ENSG00000198785, ENSG00000205562
**C4 (n=52)**	BST2, ROBO3, MGST2, EHMT2, TP53I13, RGL3, NCRNA00275, VASP, GLTSCR2, EMCN, FAM129B, TRABD, ECE1, LMNA, LRRC32, HDGF, ERG, FMNL3, GPATCH8, VWF, GJC1, IL4R, SLC25A28, RHOC, GTPBP5, COL8A1, RBMS2, BAZ2A, ARHGAP42, NACA, ABCB7, TMEM104, SOX13, SLC19A3, NOSTRIN, UBXN1, TRDMT1, RPS6, ELOVL7, CHST3, HIST1H4E, TMEM39A, RPL7, GIMAP6, MIB2, C1orf162, FAM54A, ABCA9, H2AFJ, CPSF4	ENSG00000076067, ENSG00000076108, ENSG00000077238, ENSG00000085871, ENSG00000101181, ENSG00000105373, ENSG00000109066, ENSG00000110799, ENSG00000117298, ENSG00000125753, ENSG00000130303, ENSG00000131269, ENSG00000135917, ENSG00000136830, ENSG00000137507, ENSG00000143321, ENSG00000143842, ENSG00000144810, ENSG00000154134, ENSG00000155287, ENSG00000155366, ENSG00000157554, ENSG00000160789, ENSG00000161791, ENSG00000164035, ENSG00000165895, ENSG00000167543, ENSG00000170638, ENSG00000177410, ENSG00000182963, ENSG00000186566, ENSG00000204371, ENSG00000205517, ENSG00000225196, ENSG00000235904, ENSG00000196531, ENSG00000107614, ENSG00000111332, ENSG00000122863, ENSG00000133561, ENSG00000137154, ENSG00000143110, ENSG00000146410, ENSG00000147604, ENSG00000154258, ENSG00000160917, ENSG00000162191, ENSG00000163072, ENSG00000164181, ENSG00000176142, ENSG00000197530, ENSG00000198518

Table S4. Biological processes over-represented among female-accelerated genes in the superior frontal gyrus (SFG).

The table describes the Gene Ontology (GO) biological processes and KEGG pathways over-represented among four clusters (Cluster1-4) of female-accelerated genes compared to all genes showing significant age-related expression changes, parallel expression patterns and differential expression between the two sexes (n=2490) in SFG. Cluster4 does not show significant enrichment.

[a] The number of female-accelerated genes in each cluster within the relevant GO or KEGG pathway category.

[b] The number of all other age-related and differentially expressed genes within the relevant GO or KEGG pathway category.

[c] Bonferroni corrected hypergeometric test *p*-value for the GO category/KEGG pathway enriched in female-accelerated clusters compared to other genes.

[d] This enrichment signal in pathways related to degenerative disorders arises from the same set of mitochondria-related genes associated with all three pathways (ATP5A1, COX5A, SDHB, NDUFAB1, NDUFV1, COX7A2, NDUFV3). Notably, all three disorders involve defects in mitochondrial energy production.

Gene Ontology category		#Genes in cluster[a]	#Other genes[b]	*P*-value for enrichment[c]
Cluster1 (n=198)	Regulation of neurotransmitter levels	9	12	0.002
	Cellular respiration	8	16	0.058
	Neurotransmitter secretion	6	8	0.072
	Transmission of nerve impulse	17	73	0.086
Cluster2 (n=172)	Immune system process	35	155	0.000
	Immune response	24	109	0.001
	Antigen processing and presentation	7	10	0.014
	Defense response	20	101	0.032
Cluster3 (n=232)	Nervous system development	33	156	0.013
	Regulation of cell projection organization	6	6	0.053
**KEGG pathway**		**#Genes in cluster[a]**	**#Other genes[b]**	***P*-value for enrichment[c]**
Cluster1 (n=198)	Oxidative phosphorylation	10	19	0.000
	Parkinson's disease [d]	9	18	0.000
	Huntington's disease [d]	8	27	0.018
	Alzheimer's disease [d]	8	34	0.060
Cluster2 (n=172)	Viral myocarditis	7	9	0.000
	Allograft rejection	5	3	0.001
	Type I diabetes mellitus	5	6	0.008
	Antigen processing and presentation	6	14	0.027
	Autoimmune thyroid disease	5	3	0.001
	Graft-versus-host disease	5	3	0.001
	Natural killer cell mediated cytotoxicity	7	18	0.017
	Cytokine-cytokine receptorinteraction	7	22	0.044
Cluster3 (n=232)	Neuroactive ligand-receptorinteraction	13	44	0.004

Table S5. Biological processes over-represented among female-accelerated genes in the postcentral gyrus (PCG).

The table describes the Gene Ontology (GO) biological processes and KEGG pathways over-represented among the four clusters (Cluster1-4) of female-accelerated genes compared to all genes showing significant age-related expression changes, parallel expression patterns and dimorphic expression changes between two sexes (n=2102) in PCG. Cluster1 and Cluster4 do not show significant enrichment.

[a] The number of female-accelerated genes in each cluster within the relevant GO or KEGG pathway category.

[b] The number of all other age-related and differentially expressed genes within the relevant GO or KEGG pathway category.

[c] Bonferroni corrected hypergeometric test *p*-value for the GO category/KEGG pathway enriched in female-accelerated clusters compared to other genes.

**Gene Ontology category**		**#Genes in cluster[a]**	**#Other genes[b]**	***P*-value for enrichment[c]**
Cluster2 (n=129)	Gene expression	42	360	0.01
	RNA processing	12	55	0.067
Cluster3 (n=75)	Neurological system process	14	117	0.008
	Cell-cell signaling	11	90	0.046
**KEGG pathway**		**#Genes in cluster[a]**	**#Other genes[b]**	***P*-value for enrichment[c]**
Cluster3 (n=75)	Neuroactive ligand-receptor interaction	7	38	0.001
	MAPK signaling pathway	5	40	0.041

Supporting figures

Figure S1. Age-distribution for the samples used in this study.

The x-axis shows the ages of all male and female samples across the four brain regions in DATASET1, on a linear age scale.

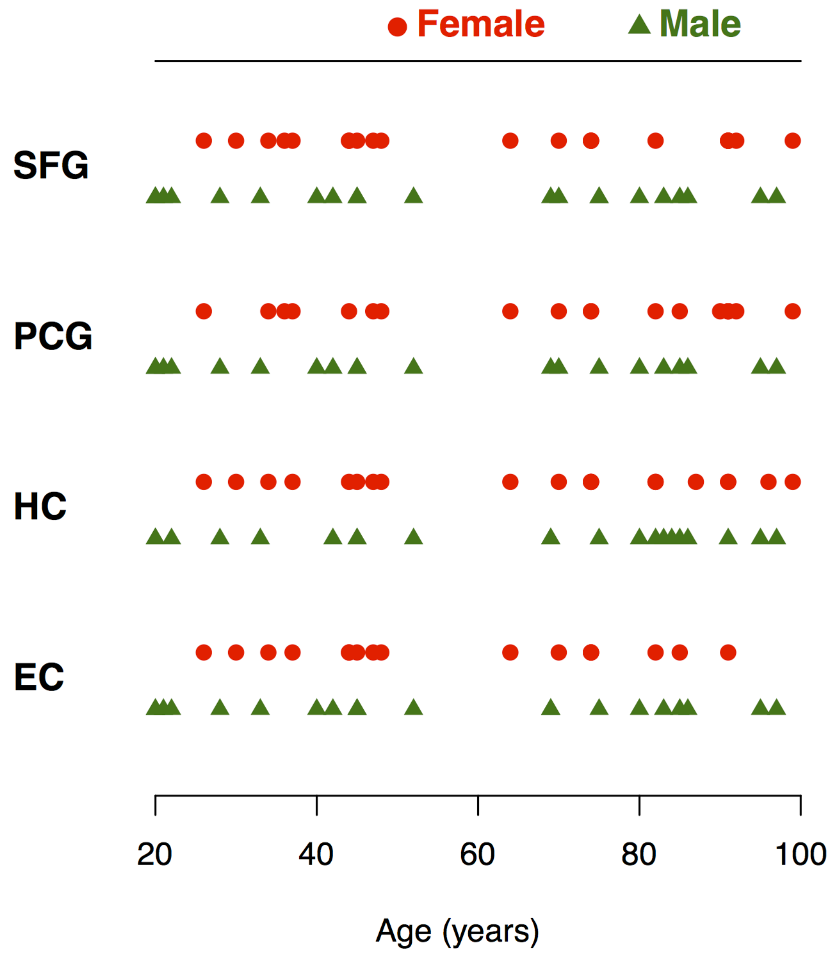

Figure S2. Sexually dimorphic expression patterns in PCG, HC and EC.

(A) Sexually dimorphic expression patterns in PCG. The six expression patterns for two sexes are shown by clustering all age-related and differentially expressed genes in PCG using non-supervised hierarchical clustering. The x-axis shows male and female age measured on a real age scale and the y-axis shows the mean expression levels, calculated after each gene's expression was converted into z-scores (mean=0, s.d.=1). The lines correspond to cubic spline curves and the vertical lines on each spline show the distribution of expression levels for individual genes within each cluster (upper 95% to lower 5%). The number above each panel is the number of genes within each cluster.

(B-C) Sexually dimorphic expression patterns in HC and EC, processed as in panel A.

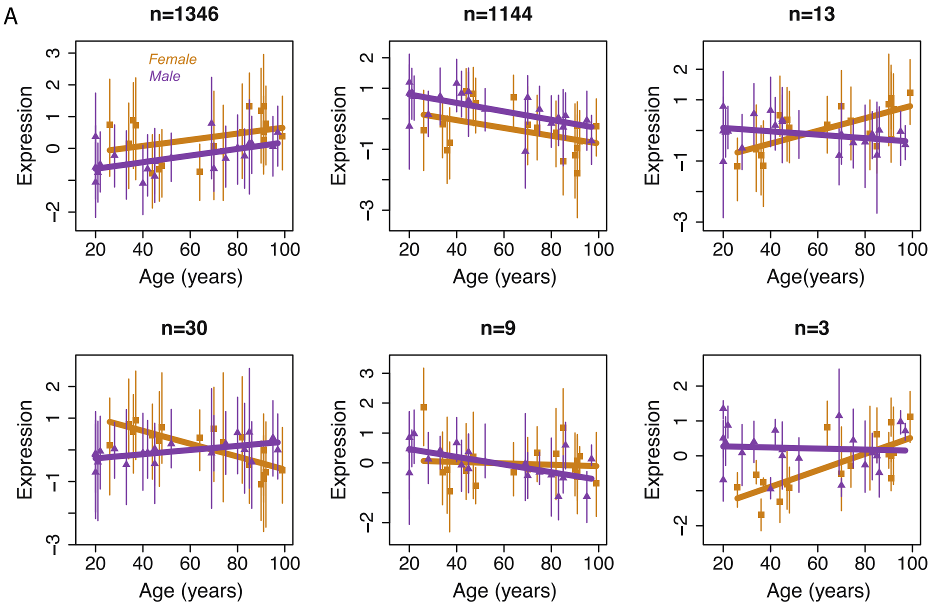

B

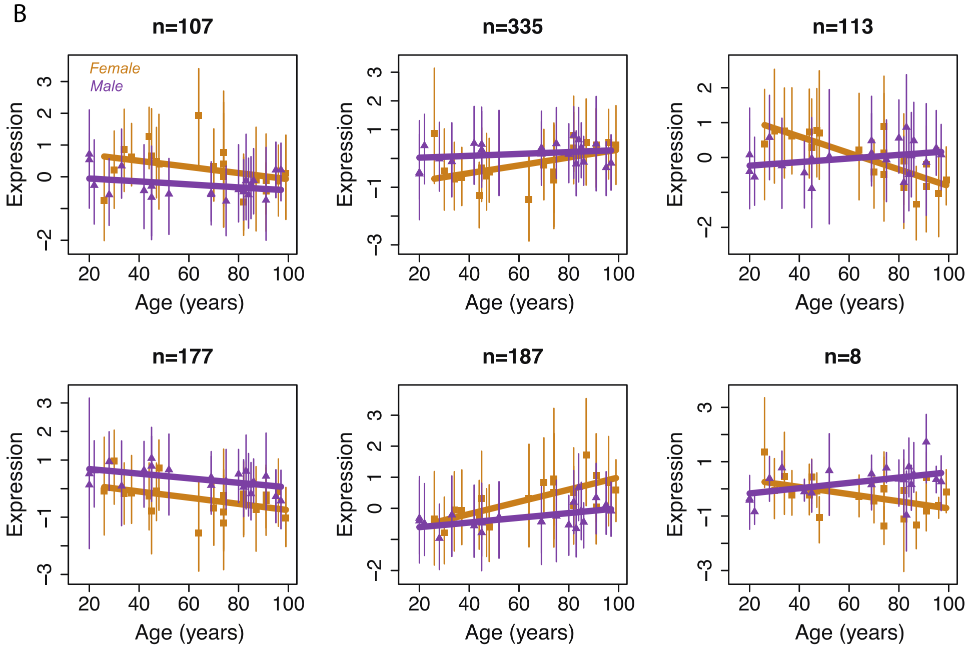
n=107
n=335
n=113
n=177
n=187
n=8
Female
Male
Expression
Age (years)

C

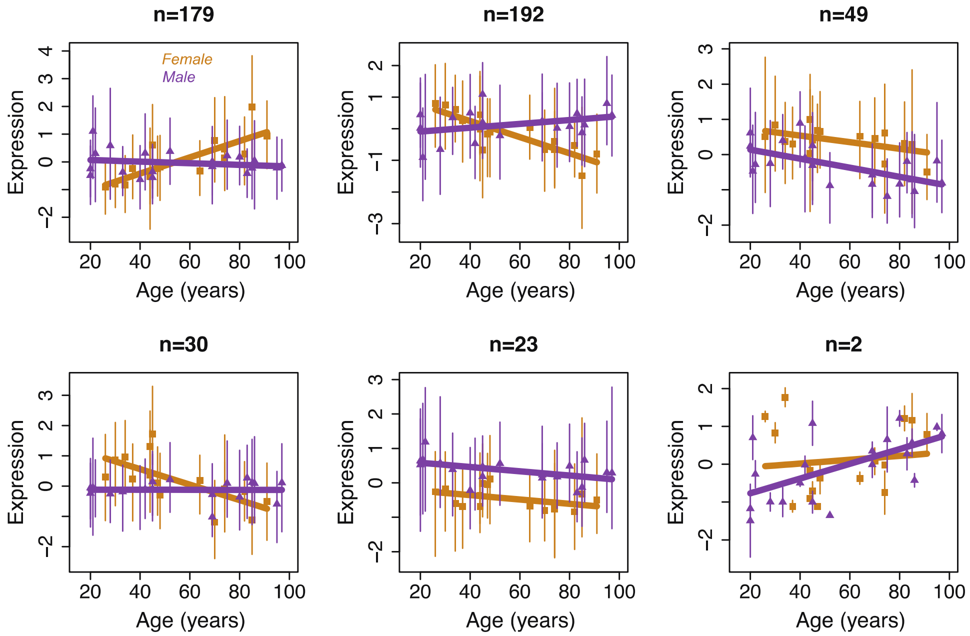
n=179
n=192
n=49
n=30
n=23
n=2
Female
Male
Expression
Age (years)

Figure S3. Two main time-shift patterns among the SFG female-accelerated genes.

Time-shifts between male and female time-series were calculated by aligning female expression data to male data, or by aligning male expression data to female data, for each gene separately (1). The numbers above each graph represent the number of genes within the cluster. The upper row panels show the mean time-shift estimates based on all genes in a cluster and the distribution of the shifts (vertical lines) for individual genes within clusters (5%- 95%). Positive values of time-shift indicate that male ages are mapped to younger ages in the female time-series, suggesting slower/delayed expression changes in males, and vice versa. The time-shift shown on the y-axis is calculated as the difference between female age and male age at aligned expression time points. The bottom row panels show the relationship between male and female ages based on the calculated time-shift estimates shown on the upper rows.

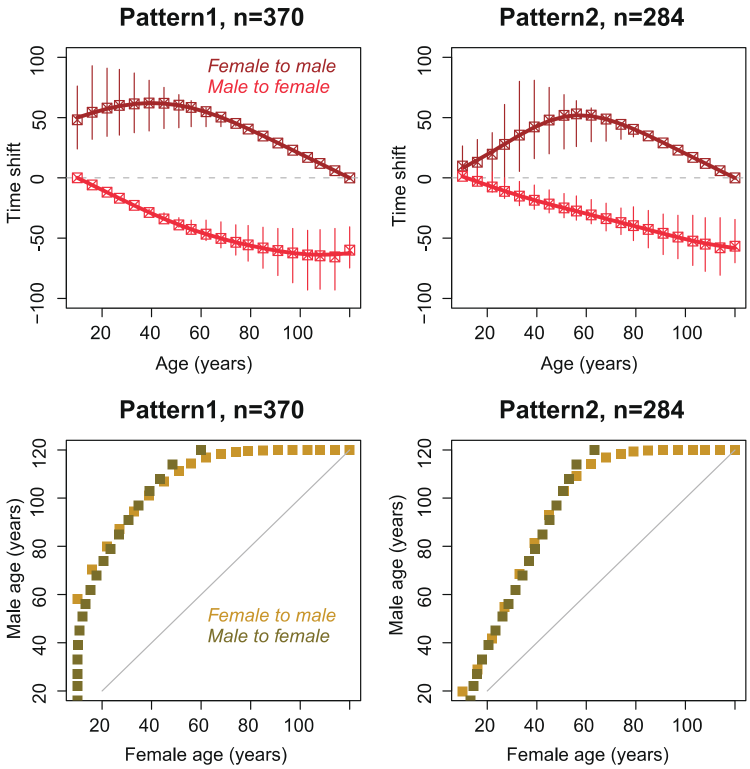

Figure S4. Expression patterns of SFG female-accelerated gene clusters in three additional datasets.

Mean expression patterns for four clusters of female-accelerated genes identified in DATASET1 (SFG), plotted using other prefrontal cortex datasets' gene expression profiles. (A) The four clusters' expression profiles in DATASET2 (PFC). The four clusters contain n=579 genes of the original 654 that were detected in DATASET2. The y-axis shows mean expression levels of genes in a cluster, after each genes' expression levels were converted into z-scores. The x-axis shows male and female age. (B) The same as in panel A, but using DATASET3 (PFC) profiles, containing n=603 genes. (C) The same as in panel A, but using DATASET4 (PFC) profiles, containing n=591genes.

Note that in some Fig S4 clusters, female and male trajectories overlap. This is especially pronounced in DATASET 2: here the signal-to-noise ratio not only for sex differences, but also for age-related change, is lower than in other datasets. However, whenever female and male trajectories do *not* overlap, and sex differences are readily detectable between trajectories, these differences are, without exception, in the direction of earlier/faster changes in females.

Also note that the clusters shown here contain the same genes (except those not expressed in a dataset) as the DATASET1 (SFG) clusters shown in Fig 2C. The genes are clustered based on their expression patterns in DATASET1, not in datasets 2-4. Consequently, their average expression profile appears noisier than those in Fig 2C.

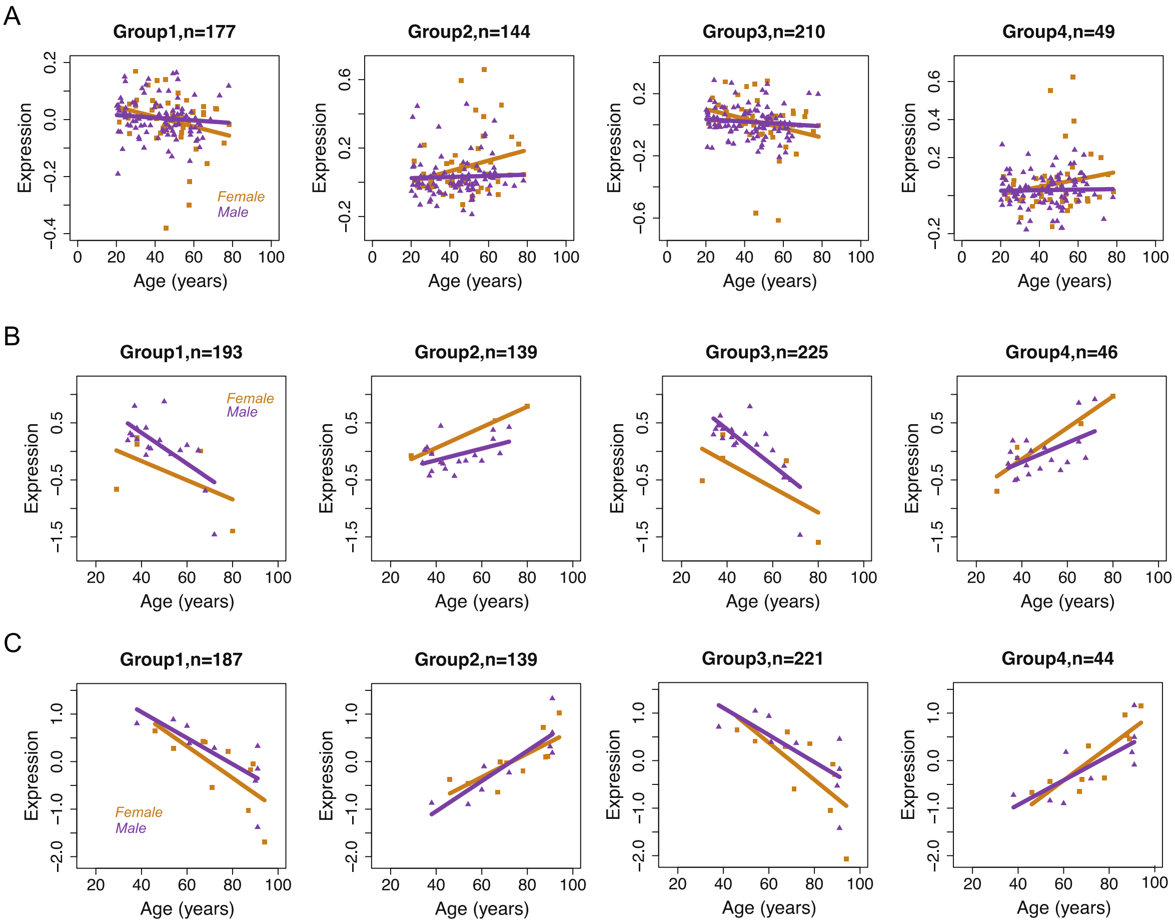
A
Group1,n=177
Group2,n=144
Group3,n=210
Group4,n=49
Female
Male
Expression
Age (years)
B
Group1,n=193
Group2,n=139
Group3,n=225
Group4,n=46
Female
Male
Expression
Age (years)
C
Group1,n=187
Group2,n=139
Group3,n=221
Group4,n=44
Female
Male
Expression
Age (years)

Figure S5. Cell type over-representation among the SFG female-accelerated gene clusters.

The graph shows the proportions of genes in each of the four SFG clusters (the 654 genes showing accelerated aging-related changes in females, and represented in Fig 2C), which overlap with genes with expression specific to a particular cell type: neurons, oligodendrocyte precursor cells (OPC), oligodendrocytes, astrocytes, or are non-specific. The cell-type-specific expression profiles were identified in (2) and the microarray data was processed as in (3).

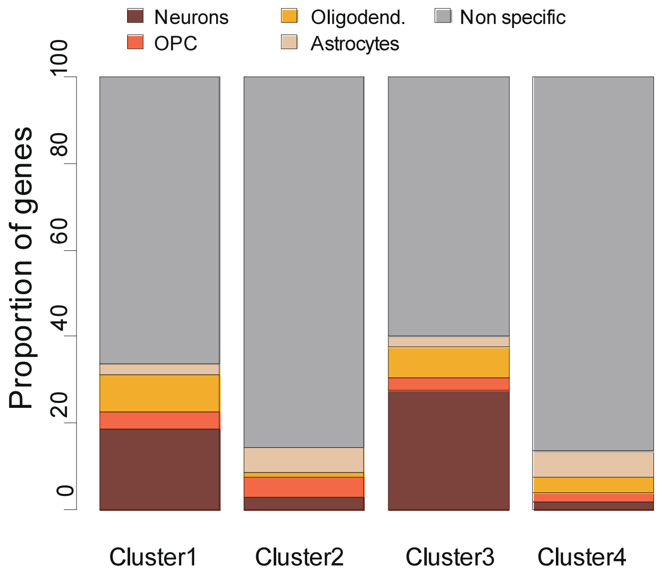

Figure S6. Expression patterns for 324 female-accelerated genes in PCG.

Genes were clustered into four groups using the same work-flow as for the 654 female-accelerated genes in SFG. The y-axis shows male and female expression levels as z-scores and the x-axis shows male and female age in years.

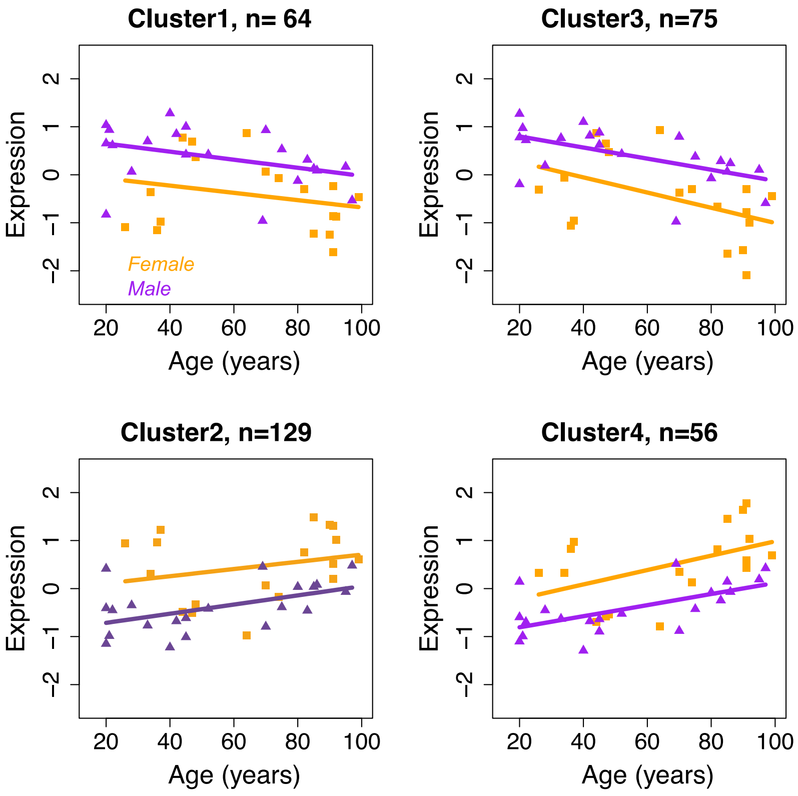

Figure S7. Expression patterns of SFG female-accelerated gene clusters in development and aging.

(A) Expression patterns for 654 female SFG accelerated timing genes (as in Fig 2C). The y-axis shows male and female expression levels as z-scores and the x-axis shows male and female age in years. Note that the clusters in Fig S7A and Fig 2B are based on exactly the same data, but were represented differently: in the former, we used cubic splines with 3 degrees of freedom (using the R “smooth spline” function) instead of linear age models. This was chosen to most readily observe average trends in the other two datasets represented in panels B and C (human and macaque brain aging, and human postnatal development, respectively), where age-related changes, especially in development, might follow non-linear trajectories. (B) Expression patterns of the four gene clusters as panel A, with gene expression profiles from a human and rhesus macaque aging dataset from (3). (C) The same as in panel B, plotted using gene expression profiles from a human postnatal prefrontal cortex development dataset (4).

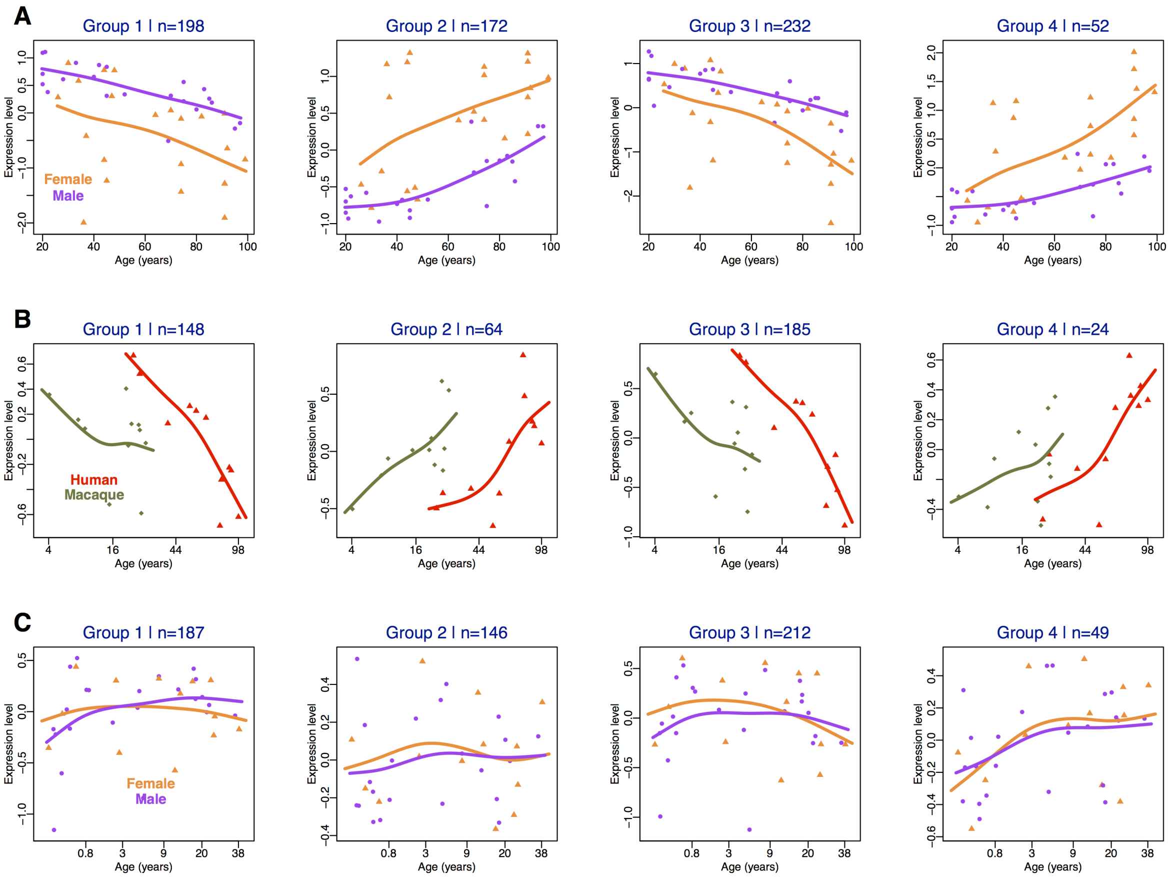

Figure S8. Clustering of individuals reveals subgrouping within females.

The graph represents a UPGMA tree based on the expression level of 654 SFG female-accelerated genes. The numbers at the nodes indicate node stability calculated by 1,000 bootstraps over genes. The node labels indicate the sex of the individuals (M or F).

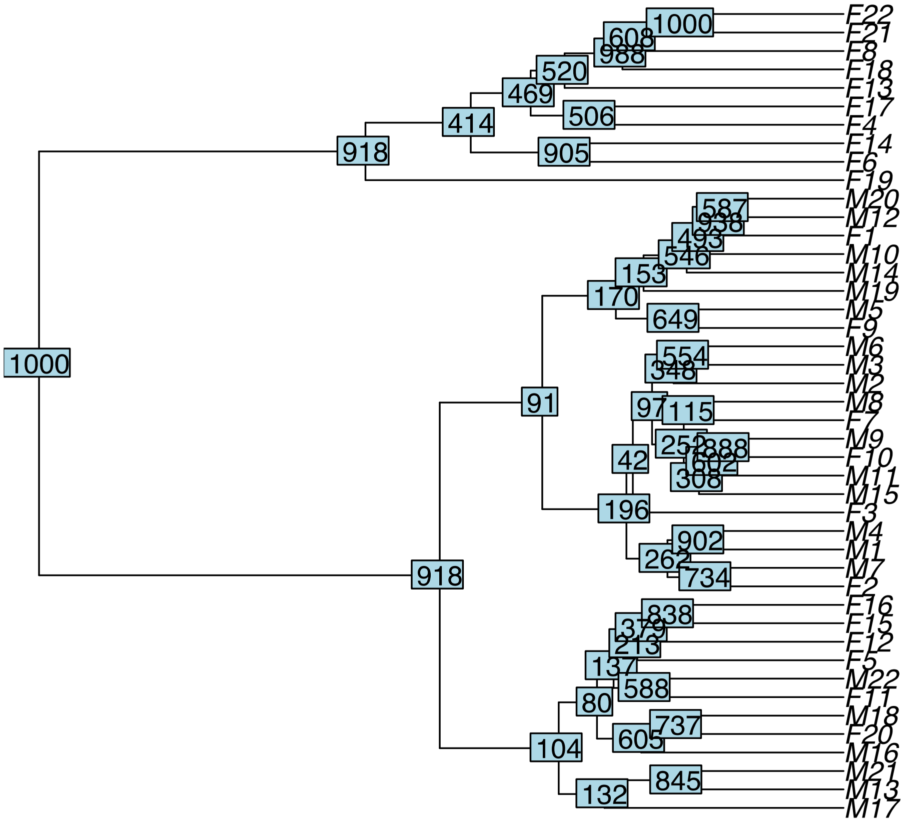

Figure S9. Sexual dimorphism in *NPY* and *NPYR* expression

Neuropeptide Y and its receptors' expression profiles in SFG. The y-axis shows the normalized expression level, while the x-axis shows individual age. Male and females are depicted in green and pink, respectively. *NPY4R* was not detected in this dataset.

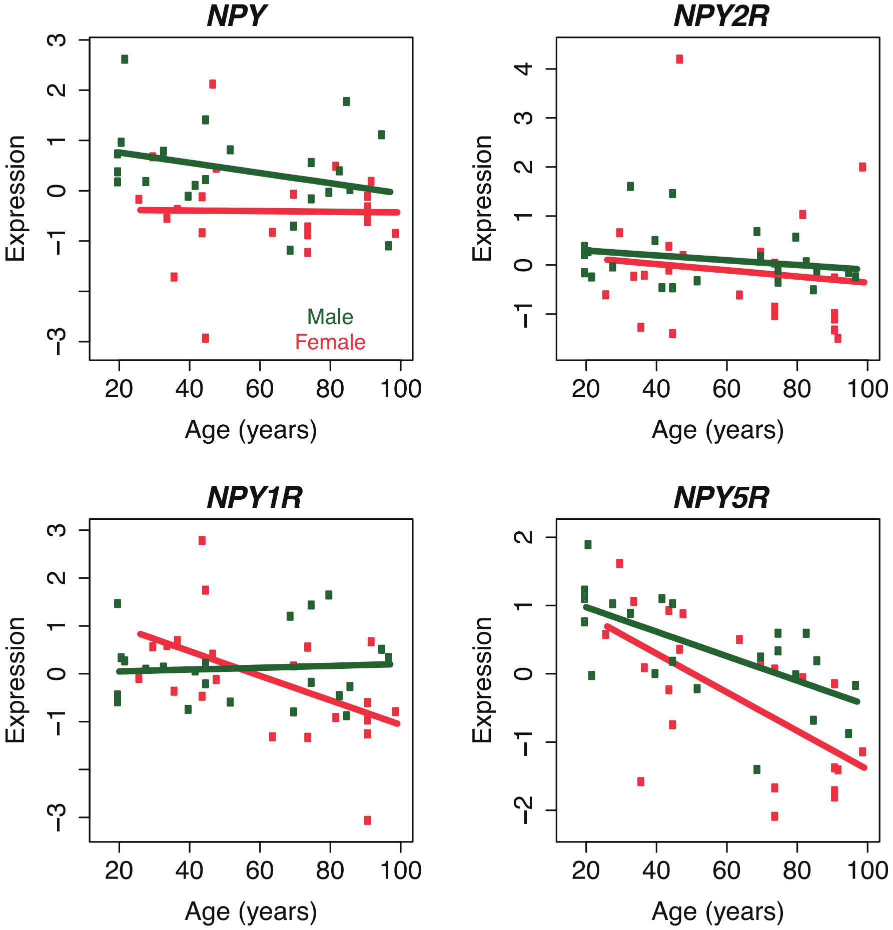

Figure S10. A.D. effect size for sexually dimorphic genes in HC.

The 584 genes showing significant sexually dimorphic expression in HC were separated into 2 groups based on their higher or lower expression in males versus females: M>F represents higher male expression in HC (n=345), and F>M represents higher female expression (n=239). The y-axis shows the corrected A.D. effect sizes after removing the age effect based on a linear regression model between A.D. effect sizes and age.

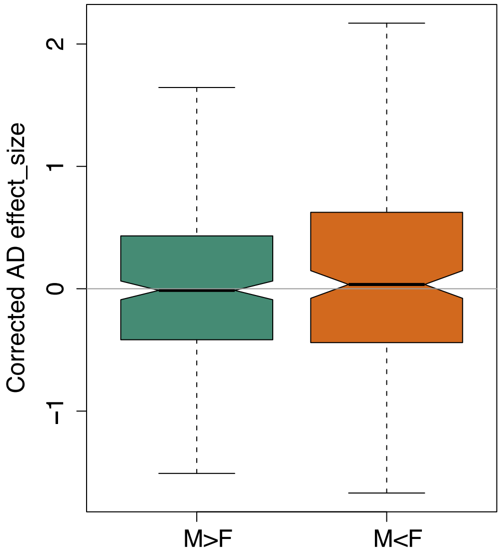

Figure S11. Male-female expression distances in faster aging and slower aging females.

F1 represents the 10 females showing faster aging patterns in SFG, determined using the bootstrap analysis shown in Fig S8. F2 are the other 12 females showing expression patterns indistinguishable from males. For expressed genes in each brain region, we calculated the absolute difference between males (M) and F1, and males and F2 (using standard normalized expression levels per gene). The boxplots show the distributions of these differences. The two distributions are significantly different in paired Wilcoxon tests ($p<0.001$), in each brain region. The effect sizes are 1.69, 1.61, 0.98 and 1.22 for SFG, PCG, HC, and EC, respectively. Note that among the 10 F1 females in SFG, only 5, 4, and 3 of the same individuals were present in the PCG, HC, and EC datasets, respectively.

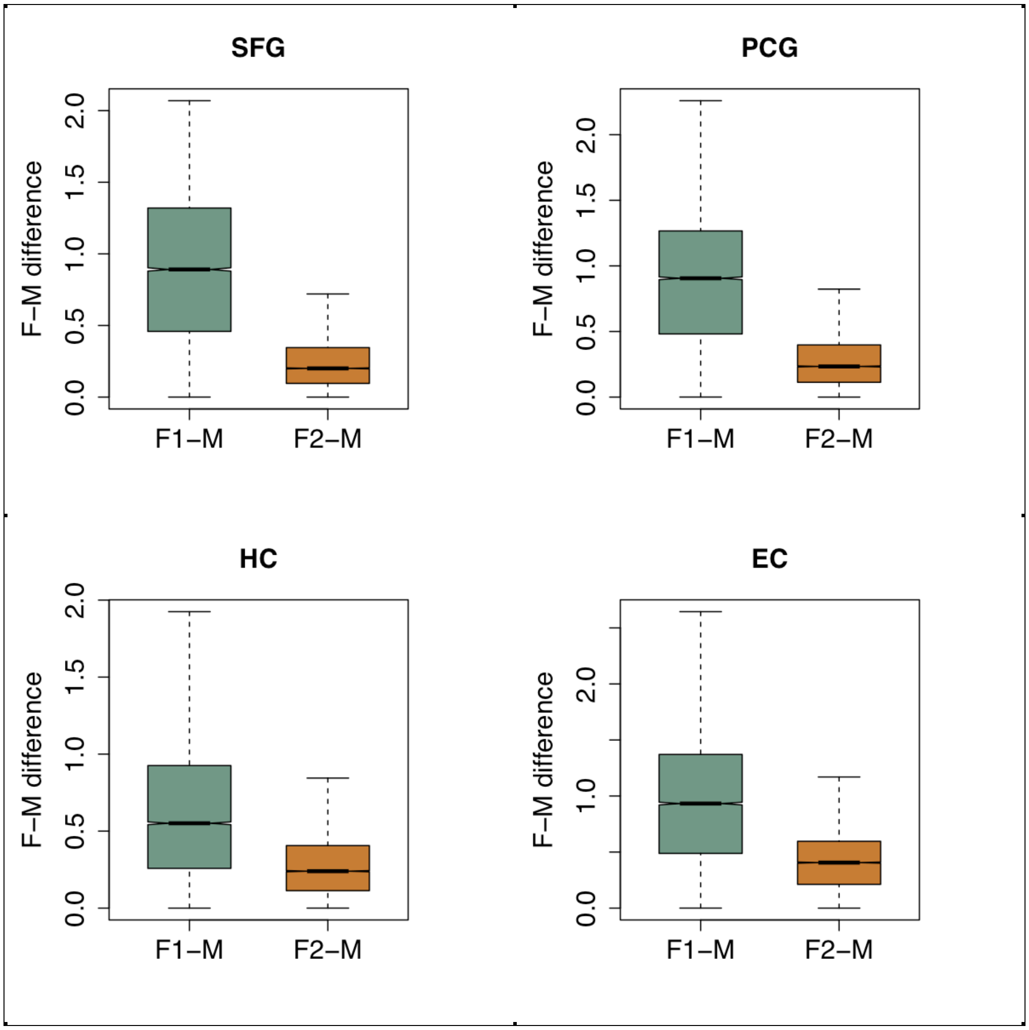

Figure S12. Number of genes showing significant sexual heterochrony in four brain regions.

The left and right panels show the number of genes with significant time-shift assigned to three categories: male-delayed (female-accelerated), female-delayed (male- accelerated), and no direction, based on aligning male expression time series to the female age scale, and aligning female expression time series to the male age scale, respectively. The number of genes shown in Fig 2A is the overlap between the two alignments.

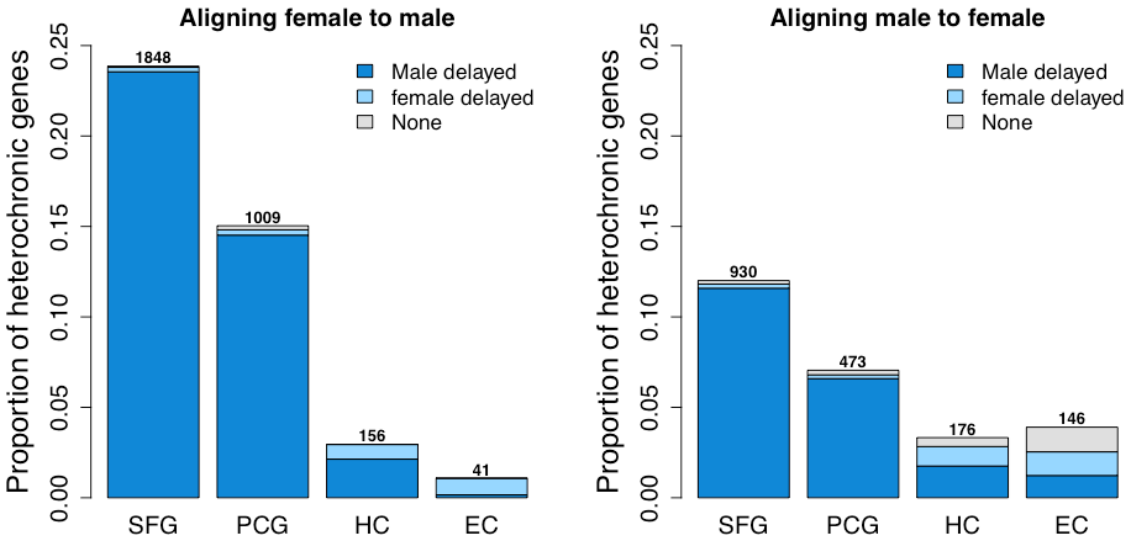

Supporting notes

DATASET1 quality control.

DATASET1 (5) was downloaded from the NCBI GEO database (http://www.ncbi.nlm.nih.gov/geo/). This is an age series based on Affymetrix HG-U133Plus2.0 microarrays, measured in four brain regions: superior-fontal gyrus (SFG), postcentral gyrus (PCG), hippocampus (HC), and entorhinal cortex (EC). In each region, the dataset contains ~20 cognitively healthy individuals for each sex, with ages ranging from 20 to 99 years (Fig S1).

We first checked sex identity by *XIST* and Y-chromosome linked gene expression, which revealed abnormal expression (too high or too low) in 10 samples (accession IDs: GSM300213, GSM300250, GSM318840, GSM300288, GSM300287, GSM300326, GSM300212, GSM300255, GSM300192, GSM300300; 4 females & 6 males). We further checked the overall variation among individuals in each region using principle components analysis (PCA) and k-means clustering, using the R "prcomp" and "kmeans" functions. This revealed 3 samples as potential outliers (accession IDs: GSM300198, GSM300301, GSM300196; 2 females & 1 male). Specifically, in k-means clustering, we found that for 400-600 genes, these samples had mean expression level >3 standard deviations distant from the average mean, whereas all other samples were within one standard deviation. We therefore chose to remove these potential outlier samples.

For the analyses presented in the main text, we preprocessed (summarized and quantile normalized) the CEL files for each brain region separately, and without including the outliers / misidentified individuals.

Spline interpolation.

For interpolation used in warping, we extended the age range of the two sexes 10-120 years. This enabled us to estimate true time-shift between males and females with greater flexibility.

Linear vs. non-linear changes

Age-related expression changes in this dataset were predominantly linear changes; nearly all these identified changes were significantly better explained by linear than non-linear

trajectories (90% in SFG, 73% in PCG, 77% in HC, 93% in EC) as measured by comparing the two models using the F-test ($p<0.05$). For this reason, we used linear models for the spline interpolation step in this dataset (d.f=2).

Positive correlation between sexes.

To ensure that age-related changes are in the same direction between sexes, we used the Pearson correlation test to compare the two sexes' expression-age trajectories ($p<0.05$).

False discovery rate estimation.

We measured the false discovery rate among the sets of age-related and differentially expressed genes using a conditional permutation. Specifically, we divided the age range into four bins of 20-40, 40-60, 60-80, 80-100 years. For each gene, we permuted the expression profile 1000 times as follows: (i) The sex labels were randomized within each age bin. (ii) Ages were permuted within each sex. (iii) The age and differential expression tests were executed. (iv) The number of both age-related and differentially expressed genes at $p<0.05$ was recorded. The median of the distribution of 'significant' genes across the 1000 permutations was defined as the expectation. FDR was defined as the ratio of this expectation to the proportion of observed significant genes (both age-related and differentially expressed genes at $p<0.05$). The FDR for the SFG was 19%, PCG 24%, EC >100%, HC 60%.

Clustering.

Genes were clustered based on their expression profiles or DTW-S-predicted time-shift profiles using the k-means and hierarchical clustering algorithms in R ("kmeans" and "hclust"). Gene expression levels were standardized to mean=0 and standard deviation=1 before clustering.

DTW-S application.

Using all age-related, differentially expressed and co-directional genes, we first aligned female expression trajectories to male trajectories, estimated the time-shift (heterochrony) between them, and conducted simulations to estimate the significance of the time-shift (1). This analysis identified 1848 genes with significant heterochrony in SFG, 1009 genes in PCG, 156 in HC, and 41 in EC (p-value cutoff for the significant time-shift points

p<0.05; (false positive rate) FPR≤17% in all four regions) (Fig S12, Table S2). We then repeated the analysis this time aligning male expression trajectories to female trajectories (Fig S12). In this comparison, 930 genes showed significant heterochrony in SFG, 473 genes in PCG, 176 in HC, and 146 in EC (FPR=12%) (Fig S12, Table S2). The difference in the number of significantly heterochronic genes identified in the two alignments (i.e. male to female, and female to male) could be caused by differences between the male and female groups' age distributions, and biological or technical noise in the expression data. We chose to be conservative and use genes showing the same type of timing difference in both alignments. We thus combined the two alignment results to generate a high confidence set of genes showing heterochronic timing between two sexes: there were 667 such genes in SFG, 336 in PCG, 81 in HC, and 35 in EC (Fig 2A).

Additional human brain aging datasets.

For analyzing sexual dimorphism in gene expression changes with age, we used three additional published datasets of the prefrontal cortex. The first (6) is based on Illumina BeadChips and contains 158 males and 73 females with ages from 0-78 years (DATASET2, with GEO ID: GSE30272). We used the data as processed by the authors, but excluding individuals younger than 20 years of age. Two other datasets were based on Affymetrix HG-U133Plus2.0 arrays. DATASET3 (7) (GEO ID: GSE21138) and contained 19 males and 5 females with ages between 29-80 years. DATASET4 (8) (GEO ID: GSE17612) contained 9 males and 10 females with ages between 38-94 years. While the original studies included more samples, we used only healthy (control) individuals, and in addition, removed potential outliers based on a PCA analysis (n=2, DATASET4). We further removed five and two male individuals from DATASET3 and DATASET4, respectively, to equalize the age range between sexes. These datasets were processed as described earlier for DATASET1. In addition, a strong batch effect in DATASET3 was removed using the ComBat algorithm (http://jlab.byu.edu/ComBat/).

Gene expression levels in all datasets were standardized to mean=0 and standard deviation=1, and we calculated average male-female differences for all the 654 genes showing significant accelerated female timing in DATASET1 SFG and detected in the respective dataset.

Further, to obtain a general impression of data quality and compatibility among the 4 datasets, we compared Pearson correlation coefficients ($r_{age\text{-}expr}$) between expression and age among genes. We calculated $r_{age\text{-}expr}$ in each dataset for each of the 528 genes with significant accelerated female timing in DATASET1 SFG that were also detected in the other datasets. We then calculated the Pearson correlation between $r_{age\text{-}expr}$ in DATASET1 and $r_{age\text{-}expr}$ in each of DATASETS 2-4 across the 528 genes. The correlations were 0.35, 0.93, and 0.75, respectively ($p<0.001$). This implies lower data quality in DATASET2 compared to the other datasets or a strong platform effect on the measure of age-related changes. This might also explain the lower overlap between sex differences DATASET2 and DATASET1.

Functional analysis.

Functional analysis of genes was conducted using Gene Ontology (GO) and Kyoto Encyclopedia of Genes and Genomes (KEGG) annotation using a Bonferroni-corrected hypergeometric test p-value cutoff ($p<0.05$) (9, 10). The Ensembl gene-GO annotation was downloaded from the Ensembl database and Ensembl gene-KEGG annotation from the KEGG database. For identifying cell type-specific expression, we used expression levels measured in the mouse central nervous system (2) and processed as described in (3). In the analyses of sexual heterochrony, we used all 2490 age-related, differentially expressed and correlated genes between the two sexes as background.

We further tested each of the four heterochronic gene clusters (Fig 2C) for enrichment in estrogen or testosterone receptor targets (using the Transfac database (11) and following the procedure in (3)), and enrichment among X chromosome-linked genes. None of these analyses yielded significant association ($p>0.05$).

References

1. Yuan, Y., Chen, Y.-P. P., Ni, S., Xu, A. G., Tang, L., Vingron, M., Somel, M., & Khaitovich, P. (2011) Development and application of a modified dynamic time warping algorithm (dtw-s) to analyses of primate brain expression time series. *BMC Bioinformatics* 12: 347.
2. Cahoy, J. D., Emery, B., Kaushal, A., Foo, L. C., Zamanian, J. L., Christopherson, K. S., Xing, Y., Lubischer, J. L., Krieg, P. A., Krupenko, S. A., *et al.* (2008) A transcriptome database for astrocytes, neurons, and oligodendrocytes: A new resource for understanding brain development and function. *J Neurosci* 28: 264-278.
3. Somel, M., Guo, S., Fu, N., Yan, Z., Hu, H. Y., Xu, Y., Yuan, Y., Ning, Z., Hu, Y., Menzel, C., *et al.* (2010) Microrna, mRNA, and protein expression link development and aging in human and macaque brain. *Genome Res* 20: 1207-1218.
4. Somel, M., Franz, H., Yan, Z., Lorenc, A., Guo, S., Giger, T., Kelso, J., Nickel, B., Dannemann, M., Bahn, S., *et al.* (2009) Transcriptional neoteny in the human brain. *Proc Natl Acad Sci USA* 106: 5743-5748.
5. Berchtold, N., Cribbs, D., Coleman, P., Rogers, J., Head, E., Kim, R., Beach, T., Miller, C., Troncoso, J., Trojanowski, J., *et al.* (2008) Gene expression changes in the course of normal brain aging are sexually dimorphic. *Proc Natl Acad Sci USA* 105: 15605-15610.
6. Colantuoni, C., Lipska, B. K., Ye, T., Hyde, T. M., Tao, R., Leek, J. T., Colantuoni, E. A., Elkahloun, A. G., Herman, M. M., Weinberger, D. R., *et al.* Temporal dynamics and genetic control of transcription in the human prefrontal cortex. *Nature* 478: 519-523.
7. Torkamani, A., Dean, B., Schork, N. J., & Thomas, E. A. (2010) Coexpression network analysis of neural tissue reveals perturbations in developmental processes in schizophrenia. *Genome Res*.
8. Maycox, P. R., Kelly, F., Taylor, A., Bates, S., Reid, J., Logendra, R., Barnes, M. R., Larminie, C., Jones, N., Lennon, M., *et al.* (2009) Analysis of gene expression in two large schizophrenia cohorts identifies multiple changes associated with nerve terminal function. *Mol Psychiatry* 14: 1083-1094.
9. Ashburner, M., Ball, C., Blake, J., Botstein, D., Butler, H., Cherry, J., Davis, A., Dolinski, K., Dwight, S., Eppig, J., *et al.* (2000) Gene Ontology: Tool for the unification of biology. *Nat Genet* 25: 25-29.
10. Kanehisa, M., Araki, M., Goto, S., Hattori, M., Hirakawa, M., Itoh, M., Katayama, T., Kawashima, S., Okuda, S., Tokimatsu, T., *et al.* (2008) KEGG for linking genomes to life and the environment. *Nucleic Acids Res* 36: D480-484.
11. Kel, A. E., Gössling, E., Reuter, I., Cheremushkin, E., Kel-Margoulis, O. V., & Wingender, E. (2003) Match: A tool for searching transcription factor binding sites in DNA sequences. *Nucleic Acids Res* 31: 3576-3579.
